# Cell-specific characterization of the placental methylome

**DOI:** 10.1186/s12864-020-07186-6

**Published:** 2021-01-06

**Authors:** Victor Yuan, Desmond Hui, Yifan Yin, Maria S. Peñaherrera, Alexander G. Beristain, Wendy P. Robinson

**Affiliations:** 1 BC Children’s Hospital Research Institute, Vancouver, BC Canada; 2 Department of Medical Genetics, University of British Columbia, Vancouver, BC Canada; 3 Department of Obstetrics and Gynaecology, University of British Columbia, Vancouver, BC Canada

**Keywords:** DNA methylation, Placenta, Human, Epigenetics, Microarray, EPIC array, Trophoblasts, Immune cells, EWAS, Pregnancy

## Abstract

**Background:**

DNA methylation (DNAm) profiling has emerged as a powerful tool for characterizing the placental methylome. However, previous studies have focused primarily on whole placental tissue, which is a mixture of epigenetically distinct cell populations. Here, we present the first methylome-wide analysis of first trimester (*n* = 9) and term (*n* = 19) human placental samples of four cell populations: trophoblasts, Hofbauer cells, endothelial cells, and stromal cells, using the Illumina EPIC methylation array, which quantifies DNAm at > 850,000 CpGs.

**Results:**

The most distinct DNAm profiles were those of placental trophoblasts, which are central to many pregnancy-essential functions, and Hofbauer cells, which are a rare fetal-derived macrophage population. Cell-specific DNAm occurs at functionally-relevant genes, including genes associated with placental development and preeclampsia. Known placental-specific methylation marks, such as those associated with genomic imprinting, repetitive element hypomethylation, and placental partially methylated domains, were found to be more pronounced in trophoblasts and often absent in Hofbauer cells. Lastly, we characterize the cell composition and cell-specific DNAm dynamics across gestation.

**Conclusions:**

Our results provide a comprehensive analysis of DNAm in human placental cell types from first trimester and term pregnancies. This data will serve as a useful DNAm reference for future placental studies, and we provide access to this data via download from GEO (GSE159526), through interactive exploration from the web browser (https://robinsonlab.shinyapps.io/Placental_Methylome_Browser/), and through the R package *planet*, which allows estimation of cell composition directly from placental DNAm data.

**Supplementary Information:**

**Supplementary information** accompanies this paper at 10.1186/s12864-020-07186-6.

## Background

A well- functioning placenta is critical for the healthy development of the fetus during pregnancy. DNA methylation (DNAm) profiling of the placenta has been increasingly used to characterize underlying processes associated with adverse perinatal outcomes (e.g. maternal preeclampsia, fetal growth restriction and preterm birth) as well as to study the influence of maternal exposures on epigenetic programming. DNAm is an epigenetic modification that can regulate or respond to changes in gene expression [1, 2]. However, because heterogeneous tissues, such as the placenta, are made up of several cell types, each with a distinct DNAm signature, whole-tissue measurements are ultimately an average of the DNAm signatures of the constituent cell types, weighted by their respective frequency in the bulk tissue sample. Therefore, changes in DNAm measured in complex tissues can often be attributed to variation in cell composition rather than DNAm changes that occur in the constituent cell populations [3]. This makes interpretation of placental DNAm studies difficult until placental DNAm is characterized at a cell-specific resolution.

During the first few cell divisions after fertilization, there is a wave of genome-wide erasure of DNAm, followed by de novo DNAm in the inner cell mass [4]. Deriving from the inner cell mass are fetal tissues and the mesenchymal core component of the placental chorionic villi (CV). Within the mesenchymal core, stromal cells (SC) and fetal macrophages called Hofbauer cells (HB) can be seen in the placental stroma as early as 18 days post conception [5], which are thought to derive from mesenchymal stem cells. HBs are distinct from decidual macrophages and fetal/maternal monocytes [6]; they display high phenotypic diversity, promoting angiogenesis early in gestation and later participating in the immune response to pathological processes and infection [7, 8]. Placental vasculature is critically important for proper functioning of the placenta, and depends on the development of vessels beneath the trophoblast layer. These vessels are formed from endothelial cells (EC) that derive from the chorionic mesoderm [9]. Encompassing the mesenchymal core is a thick trophoblast (TB) epithelial cell layer, which displays a hypomethylated profile [10]. TBs comprise a set of functionally distinct subtypes, each with their own unique function [11, 12]: Cytotrophoblasts (CTB) are stem-like cells that harbor regenerative abilities and give rise to the two major subtypes of TB, the extravillous trophoblast (EVT) and the syncytiotrophoblast (STB). EVT are motile cells that travel to maternal tissue and remodel maternal vasculature, while STB are a multi-nucleated epithelial layer lining the CV that perform critical roles in hormone production and nutrient transfer.

As a consequence of its distinct developmental origin, dramatic differences in DNAm between placenta and somatic tissues have been observed [10]. Globally, the placenta is hypomethylated compared to other tissues, which was originally attributed to reduced methylation of repetitive element DNAm [13, 14], but was later resolved to be primarily due to placental-specific partially methylated domains (PMDs) [15]. PMDs are long regions of intermediate/low DNAm surrounded by regions of higher DNAm that exist in a highly cell-specific fashion [16]. It is unclear if these PMDs have a distinct function or are footprints of earlier developmental events between embryonic and extraembryonic tissues. Parent-of origin specific DNAm, which is associated with genomic imprinting, is also more commonly found in the placenta than other tissues [17]. Almost all known imprinted genes are imprinted in the placenta, and many are exclusively imprinted in the placenta [18–21]. Interestingly, a number of placental-specific imprinted genes are polymorphically imprinted [18]. It is possible that cellular and genetic heterogeneity can contribute to polymorphic imprinting, as well as variability in DNAm generally. Supporting this, a significant role for genetic control of placental DNAm variation was recently characterized [22]. These studies have contributed to our understanding of the unique epigenetics of the placenta, but it remains unclear if these features are maintained in all constituent placental cell types or are confined to specific ones.

Placental DNAm is often studied in the context of health in relation to disease and environmental exposures. A common study design is the epigenome-wide association study (EWAS) [23], where differentially methylated CpGs (DMCs) are identified in a high-throughput manner, usually with microarray or sequencing based approaches. However, placental DNAm studies are almost all carried out using whole CV and are therefore subject to challenges of interpretability due to potential cell composition variability [24]. Unlike other tissues, such as adult blood and umbilical cord blood, addressing cell composition variability in placenta is difficult due to a lack of reference placental DNAm profiles, which enables bioinformatic estimation of cell composition from cellular deconvolution techniques [25]. These methods operate by modelling the whole tissue measurements as a weighted sum of cell type -specific DNAm signatures, where the weights correspond to the relative proportion of each constituent cell type in the whole tissue sample, and can be determined using least-squares or non-constrained regression approaches [25–27]. Without reference DNAm profiles for each cell population, researchers sometimes account for cell composition using reference-free deconvolution methods. However, the effectiveness of reference-free deconvolution in capturing cell composition variation has not yet been assessed in placenta.

To address these challenges, in this study we have generated DNAm reference profiles for 4 major human placental cell populations using the 850 k Illumina EPIC DNA methylation microarray, which profiles more than 850,000 CpGs. Our study is the first to characterize the DNAm of major placental cell populations with a high-resolution approach, across first trimester and term placentas. We show that cell-specific DNAm occurs at thousands of CpG sites, of which a subset can be used to infer cell composition using cellular deconvolution. Our study underscores the importance of cell-specific approaches in placental studies, especially when measuring epigenetic features such as DNAm.

## Results

### Major human placental cell types have highly specific methylation patterns

To characterize the dynamics of CpG methylation during human placental development, we performed microarray profiling (Illumina EPIC methylation array, n CpGs = 737,050 after removal unreliable probes) in samples of matched CV and 4 fluorescence-activated cell sorted (FACS) cell- types (Additional File 1: Figure S1A), from 9 first trimester (6.4–13 weeks gestational age) and 19 term (36.4–40.4 weeks) pregnancies (Table 1). Immunofluorescence staining of flow cytometry sorted cells (Additional File 1: Figure S1B-E) determined high purity for TB (KRT7+, 97%), HB (CD68+, 95%), and EC (CD31+, 88%) and lower purity for SC (VIM+, 73%). Several bioinformatic approaches, such as array-based sex inference [28], and genotype clustering, were used to identify contamination with maternal DNA (Additional File 1: Figures S2A-F, Additional File 2: Supplementary methods). We restricted analysis to samples with an estimated maternal cell contamination of less than 35%, with the majority of first trimester samples having less than 20%, and term samples less than 10% (Additional File 1: Figure S2G). This resulted in the exclusion of: 6 HB, 1 EC, and 4 TB from first trimester, and 1 HB from term samples. Final sample numbers in all downstream analyses are shown in Table 1.

**Table 1:** Number of cell-specific and matched chorionic villi samples from first trimester and term placentas, measured on the Illumina EPIC methylation array. Surface markers for flow cytometry and immunofluorescence staining are shown in brackets

	First trimester	Term
Chorionic villi	7	19
Trophoblast (EGFR+/KRT7+)	5	19
Hofbauer (CD14+/CD68+)	3	18
Endothelial (CD34+/CD31+)	8	19
Stromal (VIM+)	9	19
Mean Gestation age (mean and range in weeks)	10.8 (6.4–13)	39.0 (36.4–40.4)
Sex (n Males)	4	9

**Fig. 1:**
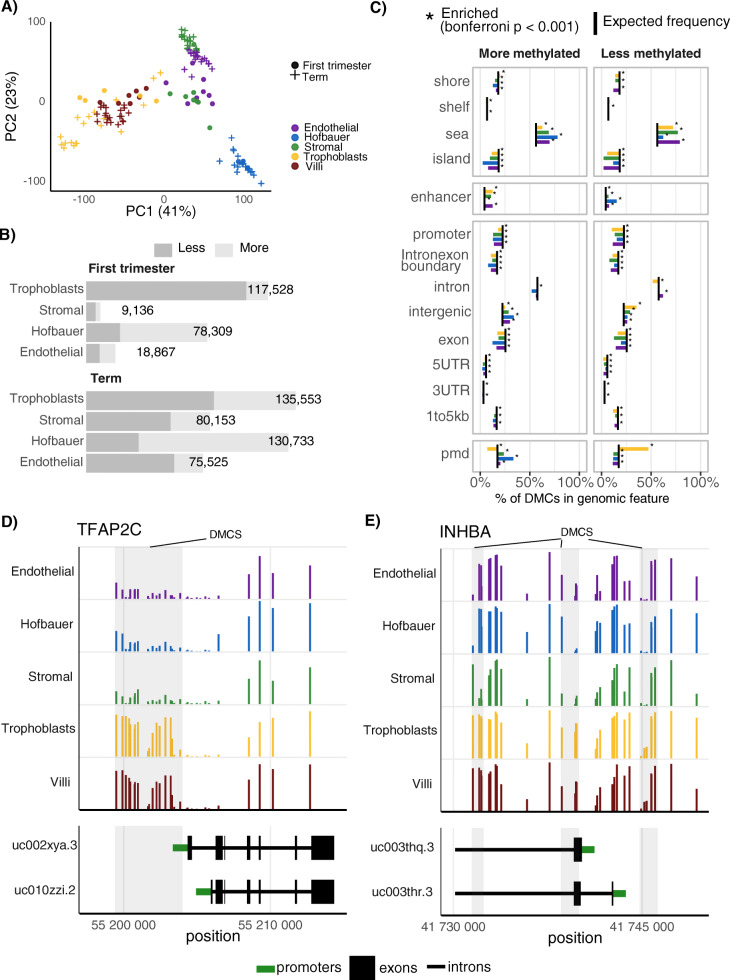
Genome-wide characterization of placental cell DNA methylation. **a** Principal components analysis (PCA) was applied to all samples and CpGs. Samples are projected onto axes PC1 and PC2 which account for 41% and 23% total variance, respectively. **b** Results from the differential methylation analysis using the R package *limma* are shown here. DMCs, defined as those tests passing a Bonferroni-adjust *p*-value < 0.01, and a difference in group means > 0.25, were divided into less methylated and more methylated compared to all other cell types. **c** Enrichment analysis of term cell-specific DMCs was carried out on genomic elements using a chi-squared test and a Bonferroni-adjusted p-value < 0.01. The expected (background) frequency, which is the percentage of total tested CpGs in each genomic element, is shown as a black line. **d** Average term placental cell-specific DNA methylation across *TFAP2C* transcripts on chromosome 6, and **e** *INHBA* transcripts on chromosome 7. Differentially methylated regions (defined as regions with a high density of differentially methylated CpGs), are highlighted with a grey background. Y axis ranges from 0 to 100% DNA methylation

To determine major factors that drive DNAm variation, we first applied principal components analysis (PCA) to all 126 CV and cell samples. Three distinct clusters were observed when samples were projected onto PCs 1 and 2 (total percent variation explained = 64%; Fig. 1a). Samples in these clusters were i) TB and CV, ii) SC and EC, and iii) HB. Cell type was strongly associated with the first 3 PCs (*p* < 0.001), while gestational age (i.e. “Trimester”) was the second strongest identifiable factor driving DNAm variation, being associated with PCs 4 and 5 (*p* < 0.001, Additional File 1: Figure S3). Technical variables such as “Batch”, “Row”, and “Chip ID” explained less variation in comparison to biological variables. Sex was associated with PCs 6 and 8–11 (*p* < 0.01). The close clustering of TB with CV (original unsorted tissue) is consistent with this being the predominant cell type in whole villi.

We next wanted to define the extent and patterns of cell-specific DNAm. At a Bonferroni-adjusted *p* < 0.01 and an absolute difference in mean methylation (Δβ) > 25%, we found 75,000–135,553 and 9136–117,528 (term and first trimester, respectively) cell-specific differentially methylated CpGs (DMCs; Fig. 1b; Additional File 3: first trimester DMCs, Additional File 4: term DMCs). The differences in the number of DMCs between first trimester (*n* = 3–9) and term (*n* = 18–19) are likely due to less power from the smaller sample size for first trimester samples compared to term. When comparing across term samples, we detected more DMCs for TB and HB (*n* = 135,553 and 130,733) compared to SC and EC (80,153 and 75,525; respectively). This was also true for first trimester samples: there were more DMCs for TB and HB (117,528 and 78,309) than SC and EC (9136 and 18,867). We further classified these DMCs by whether their methylation was in the “less than” (compared to all other cell types) or “more than” direction. Most TB DMCs were in the less methylated direction (61% - first trimester, 88% term), whereas HB DMCs were often more methylated than other cell types (74% - first, 72% term). A list of 38,656–86,355 differentially methylated regions (DMRs) were identified (FDR < 0.01) using the R package *dmrcate* for each cell type and gestational age; these results are presented in Additional Files 5 and 6.

To characterize the functional relevance of placental cell-specific DMCs, we tested these CpGs for enrichment in various genomic elements (chi-squared test, FDR < 0.05; term DMCs in Fig. 1c, first trimester DMCs in Additional File 1: Figure S4). Cell-specific DMCs were depleted in gene-related elements such as promoters, exons, 5′ UTRs, and 3′ UTRs. Instead, we saw significant enrichment in non-coding regions, such as open seas, CpG island shores, intergenic regions, introns, and enhancers. The level and direction of enrichment was highly consistent across first trimester and term cell DMCs. Less methylated DMCs were enriched for placental PMD regions [15] for TB but depleted for all other cell types. Functional enrichment analysis tested if GO or KEGG pathways were associated with cell-specific DMCs. We adjusted for the variable number of CpGs per gene to reduce bias in gene set analysis. EC and HB DMCs were enriched (FDR < 0.05) for terms related to intercellular interactions such as “cellular response to external stimulus”, whereas stromal DMCs yielded more intracellular processes related to maintaining tissue structure, such as “actin cytoskeleton” and “collagen binding”. Trophoblast DMCs were enriched for two KEGG pathways, “ECM-receptor interaction” and “Regulation of actin cytoskeleton” (Additional File 7: Table S5 and S6).

### Cell-specific DNAm occurs at highly functionally-relevant genes

A number of regions with a high density of DMCs were located in or nearby functionally- and pathology-relevant genes. *TFAP2C*, which encodes a pan-trophoblast marker, were highly methylated in TB compared to other cell types in the promoter and upstream region; whole CV showed a similar profile to TB (Fig. 1d). This region contains several predicted enhancers [29], which may require DNAm for recruiting transcription factors. Alternatively, other regions more distal to TFAP2C may be responsible for regulation of this gene’s transcription. Other trophoblast-specific markers, such as *GCM1*, *MMP2*, *SLC1A5*, and *GATA3*, also had regions of highly cell-specific DNAm localized near their transcription start sites (Additional File 1: Fig. S5). We also observed high DMC density regions in genes for which placental DNAm and/or expression differences have been associated with preeclampsia [30], including *INHBA* (Fig. 1e), *JUNB, TEAD3*, *NDRG1*, and *CGA* (Additional File 1: Figure S6). Out of 540 preeclampsia-associated CpGs previously identified by Wilson et al. 2018 that were also captured in our processed data, a statistically significant (Bonferroni adjusted *p* < 0.01) fraction ranging from 19.4–27.2% were also identified as exhibiting cell-specific DNAm for term samples (Table 2) [30].

**Table 2:** Number of preeclampsia-associated CpGs from Wilson et al. 2018 that are cell-specific DMCs for term samples. Enrichment for preeclampsia-associated CpGs was statistically significant for each term cell-specific set of CpGs at a Bonferroni-adjusted *p* < 0.01

	n cell-specific DMCs	n DMCs that are preeclampsia-associated	Proportion out of 599 preeclampsia CpGs that are also cell-specific DMCs	Odds ratio
Trophoblast	135,553	147 (0.11%)	27.2%	1.66
Stromal	80,153	105 (0.13%)	19.4%	1.98
Endothelial	75,525	109 (0.14%)	20.2%	2.22
Hofbauer Cells	130,733	131 (0.10%)	24.3%	1.49

**Fig. 2:**
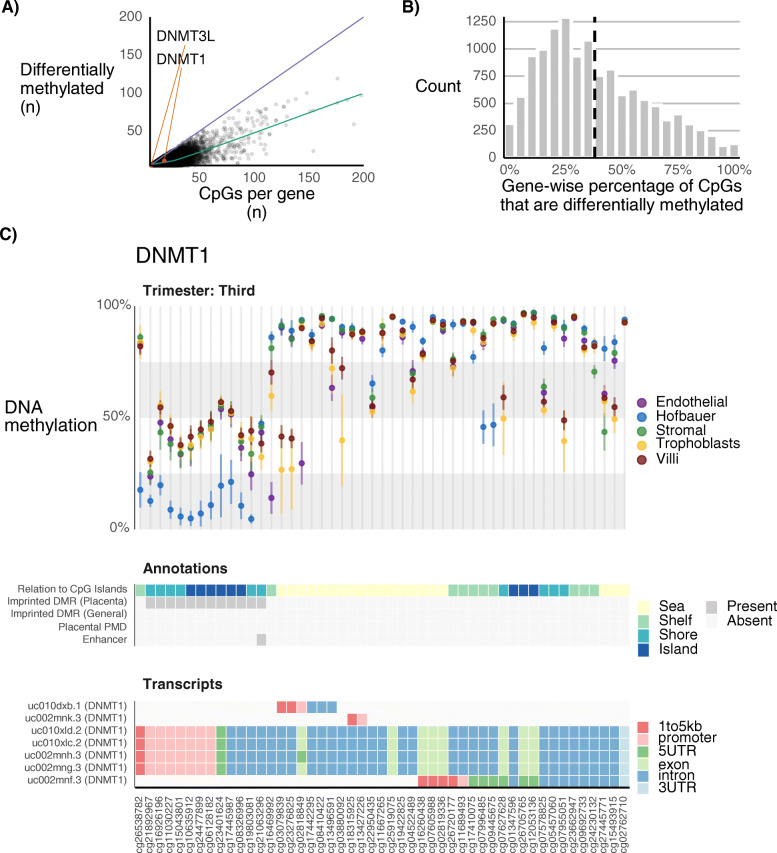
Differential methylation at DNA methylation -regulating genes. **a** On a per-gene basis, the number of promoter CpGs that are differentially methylated by at least one cell type, out of the total number of promoter CpGs per gene. The y = x line is shown (blue), where genes with 100% of promoter CpGs are differentially methylated. The green line is a smoothed average. **b** Distribution of the percentage of promoter CpGs per gene that are differentially methylated. The dotted line represents an array-wide average. **c** DNA methylation at CpGs associated with *DNMT1* for term placental samples (top). CpGs in CpG islands, imprinted regions, PMDs, and enhancers are indicated (middle). Associated UCSC transcripts and their genomic elements (promoter, 5′ UTR, exons, introns, 3’UTR) are displayed (bottom)

We hypothesized that genome-wide differences in DNAm could in part relate to differences in the expression and DNAm at genes that regulate the deposition, maintenance, and removal of DNAm, such as *DNMT1*, *DNMT3A*, *DNMT3B*, *DNMT3L*, and *TET1*. In these genes, we found that a high proportion of CpGs in the promoter region (61, 36, 31, 83, 18%, respectively) were differentially methylated by cell type. However, considering the variable number of CpGs associated with each gene’s promoter, these percentages were not significantly greater than genes of similar CpG coverage (Fig. 2 ab). Differential methylation within DNAm-regulating genes was highly localized (Figs. 2c). The promoter of DNAm-maintenance gene *DNMT1*, which is known to be specifically imprinted in the placenta [31], shows the expected intermediately methylated (i.e. ~ 50%) pattern for all cell types except HB, which is completely unmethylated (Fig. 2c). This suggests that *DNMT1* is imprinted in TB, SC, and EC, but not in HB.

### DNA methylation characterization of Syncytiotrophoblast and Hofbauer cells

**Fig. 3:**
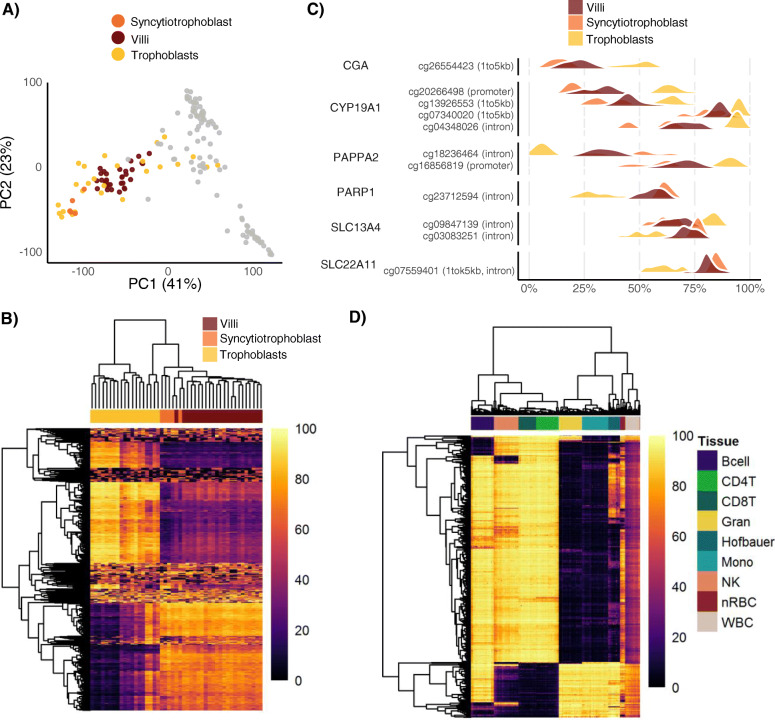
Characterization of enzymatically-separated syncytiotrophoblast and Hofbauer cell DNAm to closely related cell types. **a** Syncytiotrophoblast samples (*n* = 5) were projected onto principal components PC1 and PC2. Original samples used for constructing these PCs (Fig. 1a) are shown (chorionic villi: dark red, trophoblast: yellow, all others: grey). Syncytiotrophoblast (orange) cluster with the chorionic villi and trophoblast samples. **b** Clustering on the top 1000 variable CpGs between chorionic villi, syncytiotrophoblast, and trophoblast samples. Hierarchical clustering with Euclidean distance was used for both CpG-wise (rows) and sample-wise (columns) clustering. DNAm is shown as a range between 0 and 100%. **c** Density plots are shown for select differentially methylated CpGs, which were identified using limma, with a Bonferroni adjust *p* < 0.01, and a mean difference in DNAm > 25%. CpGs are shown along the y-axis with their locational relationship (shown in brackets) to their associated gene (left). DNA methylation is shown on the x-axis. **d** Clustering on the top 1000 variable CpGs between Hofbauer cells and cord blood cell types. Hierarchical clustering with Euclidean distance was used for both CpG-wise and sample-wise clustering. WBC: whole cord blood, nRBC: nucleated red blood cells, NK: natural killer cells, CD4T: CD4+ T cells, CD8T: CD8 T cells, Gran: granulocytes, Bcell: B cells, DNAm: DNA methylation

We used the pan-trophoblast marker EGFR to isolate TB using FACS. Because mature EVTs exist primarily in maternal tissue, and STBs are structurally incompatible with FACS isolation protocols, our TB sample likely consists primarily of CTB. In order to better understand the relationship between STB and the isolated TB cells, we compared a subset of TB with matched STB from the same placenta that was obtained from enzymatic separation using Collagenase IA (referred to as eSTB; *n* = 5) from term CV samples. This digestion protocol which extracts the outer layer of the CV, produces a sample enriched for STB, but is likely to also contain a proportion of non-STB cell types. To compare eSTB samples globally to other cell types, we projected eSTB onto PCs 1 and 2 to see where they cluster in relation to other samples. On PCs 1 and 2, eSTB clustered closely with TB and CV samples, indicating high similarity between these three populations (Fig. 3a). Throughout gestation, the STB proportion increases, and is greater in nuclei number compared to CTB at term [32]. To determine if TB or eSTB samples were more similar to CV, unsupervised hierarchical clustering was applied on the top 1000 most variable probes, and resulted in CV clustering with eSTB (Fig. 3b), which is consistent with the expectation that CV consists primarily of STB. Supporting this, we found more DMCs (Bonferroni *p* < 0.01, absolute difference in mean DNAm > 25%) between TB and eSTB (n DMCs = 4666), than between CV and eSTB (n DMCs = 72). Differential methylation at specific CpGs localized to genes known to be expressed in STB, such as *CGA*, *CYP19A1*, *PAPPA2*, *PARP1*, *SLC13A4*, and *SLC22A11* (Fig. 3c) [33–36]. The direction of DNAm at these CpGs was mostly consistent with expected patterns of genes that are more active in eSTB compared to TB and other placental cell types (i.e. more methylation at introns, less methylation at promoters).

The distinct DNAm profiles observed in placental HB suggests a distinct developmental trajectory. Indeed, the functional role and phenotypic diversity of HBs is complex and thought to vary across gestation, however, they show similar morphological and cell marker characteristics as adult and fetal monocytes [7]. Therefore, to compare placental HBs to other immune cells, we compared their DNAm profiles to a curated 450 k DNAm database of flow-sorted cord blood cell types (*n* = 263) [37]. We included only term HBs in this comparison since the available cord blood data was collected from term samples. To determine which cord blood cell types HB are most similar to, we applied unsupervised hierarchical clustering on the top 1000 most variable CpGs across each dataset. We observed that HB form their own distinct cluster (Fig. 3d), indicating they likely have unique functional properties compared to other immune cells at similar developmental stages. This finding supports previous reports of distinct DNAm between HBs, fetal/maternal monocytes, and decidual macrophages [6]. HBs cluster most closely with monocytes and granulocytes, consistent with them having a common developmental origin.

### Canonical placental epigenetic features are not always present in all constituent cells

**Fig. 4:**
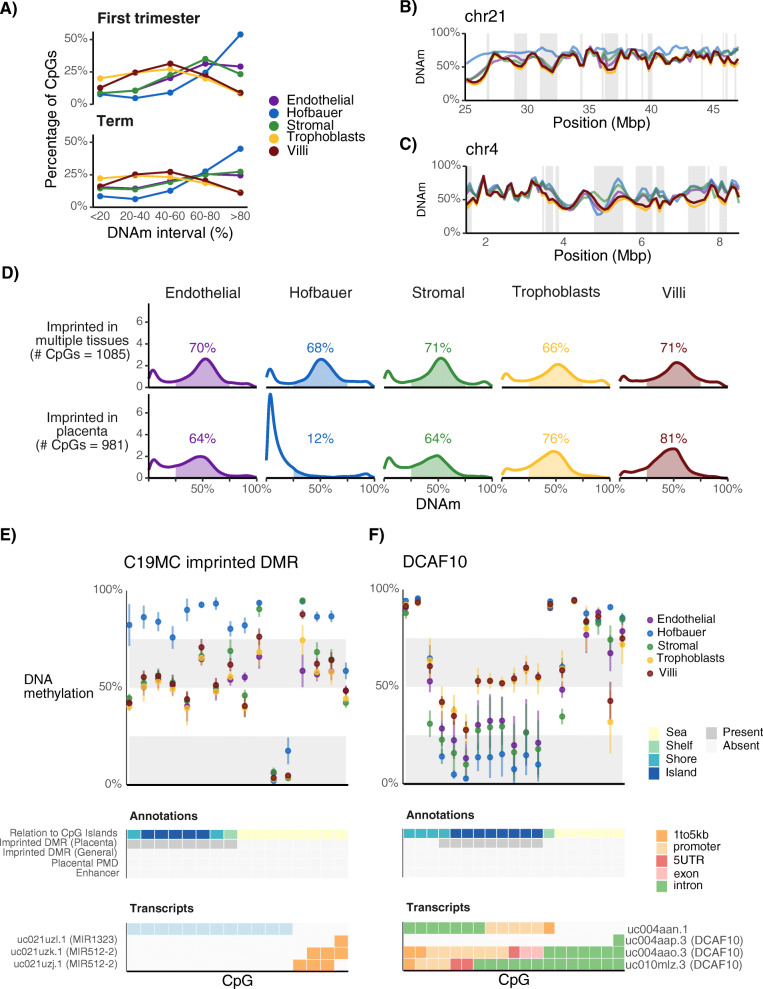
DNA methylation at partially methylated domains (PMDs), and imprinted differentially methylated regions (DMRs). **a** The percentage (y-axis) of CpGs in placental PMDs, falling into specific methylation intervals (0–20%, 20–40%, 40–60%, etc.) is shown for each cell type and trimester. **b** DNAm across specific regions on chromosome 21 (B) and 4 (**c**). PMDs are highlighted with a grey background. **d** Density plots (y-axis) of imprinted DMRs in term samples, divided into those that are imprinted in multiple tissues, (i.e. non-placental-specific; 1085 CpGs total; top) and placental-specific (981 CpGs total; bottom). The percentage of CpGs falling within 25%–75% is shown above each plot. **e** Cell-specific DNAm at the C19MC placental-specific imprinted DMR. This placental-specific imprint overlies a CpG island upstream of the miRNA cluster. **f** DNAm at placental-specific imprinted region for DCAF10

To determine if previously identified placental specific features of DNAm are cell specific, we compared cell-type specific DNAm at partially methylated domains (PMDs), genomic imprinting, and repetitive elements [15, 18, 38]. PMDs are large (> 100 kb) regions of lower average methylation (< 70%) compared to surrounding regions. Placental PMDs are thought to contribute to the observation that placental DNAm on average is much lower than other human tissues [10]. To characterize their cell-specificity, we calculated the percentage of CpGs that are found in previously defined placental PMDs [15] with DNAm falling into 20% intervals (0–20%, 20–40%, 40–60%, 60–80%, 80–100%). We observed that DNAm levels in PMDs is highly cell-specific (Fig. 4a). TB, like CV, have more CpGs with low levels of DNAm in PMDs (0–40%) compared to other cell types. HB show a strong bias towards higher DNAm levels, with over 43% of CpGs in PMDs exhibiting > 80% DNAm. We observed some changes within cell types between trimesters. All cell types have lower levels (0–40%) of methylation in term compared to first trimester. All cell types except TB have less intermediately (40–60% intervals) methylated CpGs at term compared to first trimester. HB, in contrast, have more intermediately (40–60% intervals) methylated CpGs in third trimester. In summary, the methylation levels at CpGs in PMD regions were at the expected levels (relatively low methylated compared to surrounding regions) for CV and TB; sometimes hypermethylated for EC and SC; and were almost always highly methylated for HB, at levels typically found in somatic cells.

In examining specific regions containing PMDs, a strong bimodal pattern of methylation was observed, where regions of lower methylation (overlapping known placental PMD regions), which were surrounded by regions of higher methylation (Fig. 4 BC). TB DNAm levels followed closely the levels measured in CV, supporting that placental PMDs are likely reflecting mainly TB-specific DNAm patterns. In contrast, DNAm in HB often deviated from the other cell types, typically showing higher levels of methylation within PMDs. SC and EC often “followed” CV DNAm levels, but were not nearly as consistent as TB cells in this respect.

We also looked at imprinted differentially methylated regions (DMRs) that are covered by the EPIC array. While many imprinted DMRs are maintained in somatic tissues, others are highly specific to the placenta [18–21]. To evaluate whether placental-specific imprinting is maintained in constituent placental cell populations, we first combined the results from four studies [18, 19, 21, 39] to form a list (Additional File 7: Table S7) of placental-specific (n CpGs = 981; n genes = 111) and non-placental specific (i.e. imprinted in other tissues) DMRs (n CpGs = 1085; n genes = 307). To determine if CpGs were intermediately-methylated, as would be expected for an imprinted DMR, we counted the proportion of CpGs with an average DNAm across both alleles that were in a range between 25 and 75% methylation. For CpGs in non-placental specific imprinted DMRs, the mean percentage of CpGs in the intermediate range across each cell type and in CV in term samples was 69% (Fig. 4d). For placental-specific imprinted CpGs, the percentage of CpGs falling into this DNAm range was much more variable. As expected, in the term placental samples, TB and CV had a high percentage (76, 81%, respectively) of CpGs in this DNAm range. SC and EC had a lower, but still a majority, percentage of CpGs in this range (64, 64%, respectively). In contrast, HB cells had almost no CpGs (12%) in this range; almost all CpGs were unmethylated (< 25%). These proportions were similar in first trimester samples, except with EC and SC showing less intermediate methylation and more CpGs with less methylation at placental-specific imprints (Additional File 1: Figure S7A). These results suggest that placental-specific imprinting is maintained primarily in TB, and to a lesser degree EC and SC, and is virtually absent in HB. When considering the parental origin of imprinted DNAm [18–21, 39], paternally- methylated regions had more CpGs falling within 25–75% as compared to maternal ones (Additional File 1: Figure S7BC). We only estimated this in non-placental specific imprinted DMRs, since almost all validated placental-specific imprinted DMRs are maternally methylated.

DNAm at specific imprinted DMRs was examined. As described above, TB and CV had intermediate (> 25, < 75%) DNAm at nearly all CpGs located in placental-specific imprinted regions (Fig. 4d). Most of these CpGs, in contrast, are hypomethylated for HB cells, consistent with this cell type having a different developmental origin than other placental components (embryonic versus extraembryonic). However, at the imprinted DMR associated with the placental-specific expressed microRNA cluster C19MC, this pattern is reversed: HB have hypermethylation at this region (Fig. 4e) as is reported for somatic adult/fetal tissues [18]. For SC and EC, these cell types generally show lower levels of DNAm than TB/CV at the placental-imprinted DMRs, sometimes matching that in HBs and other times showing levels somewhere between HB and TB/CV. Such patterns are observed for genes such as *DCAF10* (Fig. 4f), fibroblast growth factors *FGF8*, *FGF12* (Additional File 1: Figure S8AB), and at epigenetic regulator *JMJD1C* (Additional File 1: Figure S9A). However, for a few DMRs, levels of DNAm in SC/EC matched that of TB/CV, such as ones associated with the DNAm maintenance gene *DNMT1* (Fig. 2c) and *FGF14* (Additional File 1: Figure S9B). Higher DNAm than TB/CV was only observed for 1 gene (*RASGRF1*, Additional File 1: Figure S10).

DNAm at repetitive elements, such as Alu and LINE1 elements, can be placental-specific and have been hypothesized to often be important regulatory components of placental processes [40]. To determine if DNAm at repetitive elements is consistent across placental cell populations, we analyzed the subset of 850 k CpGs that map to Alu (*n* = 15,289) and LINE1 (22,006) elements. Compared to CV, TB had lower LINE1 DNAm (mean difference in DNAm = − 1.5%, *p* = 0.04), and HB had much higher DNAm (+ 9.7%, *p* < 0.001; Additional File 1: Figure S11A). Similar relationships are seen for Alu elements (Additional File 1: Figure S11A). TB had lower (− 1.2%, *p* = 0.02), HB had higher (+ 7.0%, p < 0.001), and EC had higher (+ 2.1%, *p* < 0.001) DNAm in Alu CpGs, when compared to CV. To explore large-scale DNAm differences, we averaged DNAm across all 850 k probes and compared each cell type to CV. We found these relationships to be similar to those with the subset of repetitive elements probes (Additional File 1: Figure S11A). HB had higher DNAm compared to CV (+ 5%, p < 0.001), and all other cell types had lower DNAm (Additional File 7: Table S8). The relationships we found for repetitive elements and global DNAm between cell types and CV were also largely consistent in our first trimester samples (Additional File 7: Table S8, Additional File 1: Figure S11B). To determine genome-wide repetitive element DNAm, we used the random forest -based ‘REMP’ algorithm [41] to predict 438,664 Alu CpGs and 39,136 LINE1 CpGs that are not covered by the EPIC array. Relationships between cell types and CV for predicted and non-predicted repetitive elements were mostly the same, except TB DNAm in predicted Alu and LINE1 CpGs was not significantly different compared to CV (Additional File 7: Table S8, Additional File 1: Figure S11C).

### Cell-specific DNAm dynamics across gestation

**Fig. 5:**
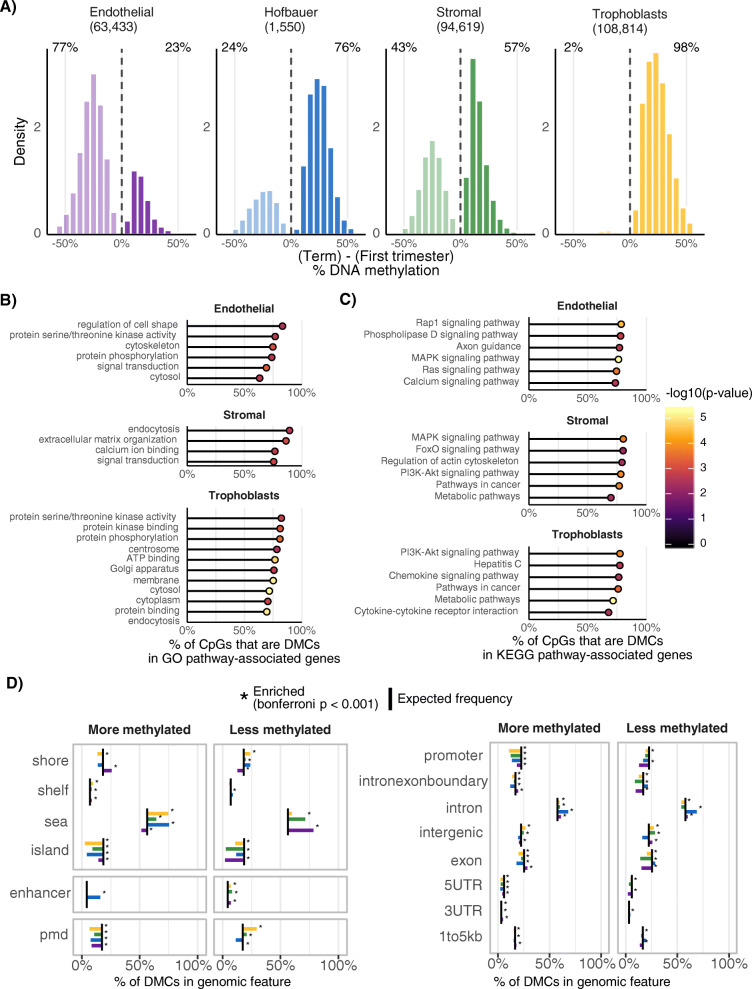
Gestational-age dependent DNA methylation within each placental cell population. **a** The distribution of the changes in DNA methylation between first and third trimester, within each cell type. Only statistically significant (Bonferroni p < 0.01) and biologically relevant (mean change in DNAm > 5%) differences are shown. Number of gestational age associated DMCs are labelled above each plot. **b** Functional enrichment analysis for GO terms tested with the R package *missMethyl*. **c** Functional enrichment analysis for KEGG pathways tested with the R package *missMethyl.* D) Enrichment for genomic features: CpG island-related elements, enhancers, PMDs, and gene features

To determine how DNAm changes in placental cell populations over gestation, we compared first and third trimester cell samples at 737,050 CpGs. We found 108,814 (TB); 94,619 (SC); 63,433 (EC) and 1550 (HB) significant cell-specific gestational-age dependent DMCs (Bonferroni *p* < 0.01, Δβ > 0.05). Strikingly, almost all of the TB DMCs show an increase in DNAm from first trimester to term (98.2%; Fig. 5a). Most gestational-age DMCs for HB and SC also show an increase in DNAm from first trimester to term (75.6 and 56.6%, respectively). In contrast, EC DMCs show less DNAm in the term compared to first trimester (77.1%).

Several interesting KEGG pathways and GO terms were significant (FDR < 0.05) in our functional enrichment analysis (Fig. 5 BC). Immune pathways (“Cytokine-Cytokine receptor interactions”) and metabolism-related terms (“metabolic pathways”, “ATP binding”, “kinase activity”) for trophoblast gestational-age dependent DMCs suggest a highly active state throughout gestation affecting multiple placental functions. As expected, stromal terms were highly associated with cellular/tissue structure -related terms, such as “extracellular matrix organization”, and “Regulation of actin cytoskeleton”. No significant pathway or GO terms were found significant for HB gestational-age DMCs. Most gestational-age dependent DMCs were enriched with open sea regions, regardless of direction of methylation (Fig. 5d). HB DMCs that increase in methylation with gestational age were the only cell type DMCs that were heavily enriched for enhancers (Bonferroni *p* < 0.001). Trophoblast DMCs that increase in methylation with gestational age were enriched for CpG island shores, open seas, and intergenic regions (Bonferroni p < 0.001). All cell type-specific gestational-age dependent DMCs were depleted (Bonferroni p < 0.001) for promoter regions, suggesting that genome-wide promoter DNA methylation is mostly stable from first trimester to term.

### Assessing cell composition in chorionic villi

**Fig. 6:**
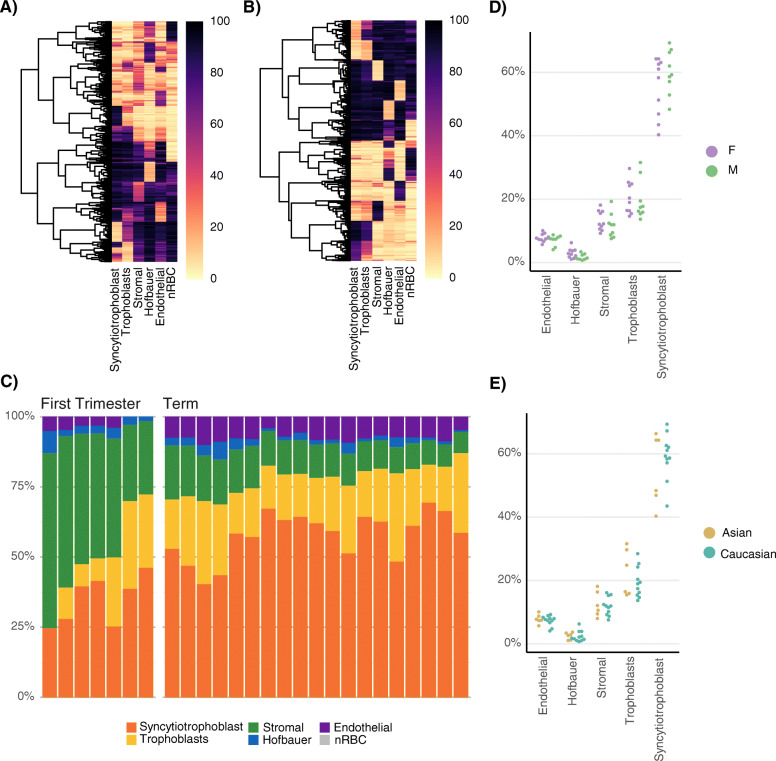
Assessing cell composition in first trimester and term CV samples. **a** Mean DNAm across each cell type (columns) for 600 first trimester deconvolution reference CpGs selected by minfi::pickCompProbes. CpGs (rows) are hierarchically clustered using euclidean distance. **b** Term reference CpGs. **c** Cell composition of 7 first trimester and 19 third trimester CV samples, estimated with cellular deconvolution using RPC. **d** Cell composition is similar between male and female term samples with respect to estimated percentage of each cell type (y-axis). F: female, M: male. **e** Cell composition is similar between Asian and European/Caucasian third trimester samples

Using placental cell DNAm profiles as a reference, we assessed cellular composition in CV samples using cellular deconvolution. To select cell-type discriminating CpGs, the *pickCompProbes* function from the R package *minfi* [42] was used, which takes the top 100 most hypo- and hyper-methylated CpGs ranked by F-test statistic for each cell type. Gestational-age specific references were created for first trimester and term. For first trimester samples, reference probes were selected from all first trimester cell samples, but also term nucleated red blood cells (nRBCs) and eSTB samples were used since these cell types are also present in early gestation [43]. For nRBC samples, 11 DNAm profiles from umbilical cord blood from public databases were included [37]. Reference CpGs determined from first trimester (Fig. 6a) and term (Fig. 6b) placental samples were highly cell-specific (Additional File 7: Table S9 and S10 for first trimester and term respectively).

To determine the best-performing cellular deconvolution method, 1500 in silico bulk mixtures were generated based on our cell data with known cellular composition proportions. These deconvolution methods were compared: constrained projection (CP) [26], robust partial correlations (RPC) [25], and support-vector regression / CIBERSORT (CBS) [27]. All three methods were tested using the implementation from the R package *EpiDISH* [44], and the constrained projection approach was used from implementations in both *EpiDISH* and *minfi* [42] R packages. Performance was high and consistent across algorithms and cell types (*R*^2^ = 0.88–0.99, RMSE = 0.02–0.08, MAE = 0.01–0.4; Additional File 1: Figure S12A; Additional File 7: Table S11). However, RPC slightly outperformed other approaches (R^2^ = 0.96, MAE = 0.024, RMSE = 0.045). Biases towards under−/over- estimation for certain cell types were small but were consistent across algorithms (Additional File 1: Figure S12B): SC tended to be overestimated (mean difference between estimated and actual = + 0.33% to 0.98%), HB were underestimated (− 0.03% to − 0.38%). TB were underestimated (− 0.07% to 0.94%), and nRBCs do not show as much bias (+ 0.03 to − 0.21%).

To assess the validity of placental cell deconvolution estimates, we applied deconvolution to previously published placental samples that are enriched for specific cell populations. Deconvolution was applied to cultured trophoblast samples (*n* = 90) from Yuen et al. 2013 [12], that were cultured to 24 h (predominantly CTB phenotype) or to 48 h, after which many CTB cells have fused into STB; each set of samples was also subjected to varying oxygen levels (1%, 8%, 20%). Cultured STB had higher estimated STB relative to sample-matched cultured CTB (Fig. S13A). The small changes in STB:CTB between culturing times are consistent with the small DNAm differences that were reported in Yuen 2013 [12], and suggest that although fusion of cytotrophoblast was achieved, further culturing would be required to produce a mature STB phenotype akin to term placenta. We then applied deconvolution to first, second trimester, and term enzymatically separated mesenchyme (*n* = 3) and matched samples of outer TB layer of chorionic villi (*n* = 3) from Hanna et al. 2013 [18], the latter of which were isolated in the same manner as eSTB in the present study. Despite batch effects and array differences (450 k vs 850 k), the term TB sample was estimated to be mostly syncytiotrophoblast (97%; Additional File 1: Figure S13B). Deconvolution estimates for trophoblast isolated from first and second trimester placentas were also mostly TB with some presence of the mesenchymal components, in particular some SC. Matched mesenchyme samples, as expected, were enriched for SC, and EC. Overall, these findings are consistent with our understanding that enzymatic separation enriches for certain populations but cannot produce homogenous cell populations. Lastly, we applied cell deconvolution to chorionic villi samples (*n* = 5) that were enriched for large visible stem villi. These samples had cell compositions that were heavily enriched for SC (mean = 51%, sd = 4%), compared to matched “normally” -processed chorionic villi (mea*n* = 11%, sd = 2%; Additional File 1: Figure S13C).

RPC cellular deconvolution was applied to our 7 first trimester and 19 term CV samples. There was significant gestational-age specific variation in the estimated percentage of eSTB, TB, and SC (Table 3; Fig. 6c). eSTB were the most abundant cell type in all (19/19) term samples (mean = 58%), whereas SC was the most abundant in most (5/7) first trimester samples (mean = 43%). There were significant changes from first trimester to term samples: there was a significant mean increase of 23% in eSTB (Bonferroni-adjusted *p* < 0.001), a decrease in SC (− 31%; adjusted *p* < 0.001) and a small increase in EC (+ 5%; adjust *p* < =0.005). A detectable contribution of nRBCs was not estimated in any sample using RPC deconvolution. No significant (adjusted *p* > 0.01) differences in cell composition were observed between male (*n* = 9) and female (*n* = 10) samples (Fig. 6d; Additional File 7: Table S12), or between European/Caucasian (*n* = 11) and East Asian (*n* = 6) samples (Fig. 6e; Additional File 7: Table S12) for term CV. Within-trimester gestational age (estimated and reported) was not significantly associated with cell composition (Additional File 7: Table S12), although numbers were small.

**Table 3:** Mean of cell composition estimates (%) for first trimester and term CV samples using RPC cellular deconvolution. Standard deviation is shown in parentheses

	First (***n*** = 7)	Term (***n*** = 19)
Syncytiotrophoblast	35 (9)	58 (8)
Trophoblast	16 (12)	20 (6)
Stromal	43 (13)	12 (3)
Hofbauer cells	3 (2)	2.34 (1)
Endothelial	3 (2)	7 (1)
Nucleated red blood cells	0 (0)	0 (0)

## Discussion

We performed a comprehensive analysis of DNAm for human placental cell types using the Illumina EPIC methylation array. Previous placental cell DNAm studies have focused on a lesser number of cell types [45], used lower resolution approaches [46], or focused on a narrow gestational age range (e.g. only first trimester, or only term). Using the EPIC array, which targets CpG sites in gene-rich regions and non-coding regulatory elements, this study describes the DNAm profiles of major human placental cell types from first trimester and term placentas, and identifies cell-specific and gestational age –dependent DNAm.

After the wave of de novo DNAm in the inner cell mass and trophectoderm, global differences in DNAm exist between these two blastocyst cell layers and their derivatives. These differences result in genome-wide patterns with the placenta showing a unique hypomethylated DNAm profile compared to other somatic tissues [47]. Earlier studies suggested that the hypomethylated placenta was partly due to lower DNAm at repetitive elements such as LINE1 [13, 14, 40]. We show that LINE1 and ALU DNAm is higher in HB compared to other placental cell types, but otherwise displays low cell-specificity. Later studies indicated that placental hypomethylation could be largely attributed to long regions of consistently low methylation (PMDs), and that this type of patterning was unique to the placenta [15]. We found that PMDs are more pronounced in TB, and are absent from HB. The impact of PMDs is unclear and may in part reflect that in the blastocyst, the trophectoderm does not undergo de novo DNAm. Whether PMDs serve a functional role in the placenta is also unclear, but our understanding of their relevance would benefit from characterizing the timing of their development. We note that although our study is genome-wide, the number of CpG loci analyzed (*n* = 737,050) is only a fraction of the epigenome (~ 3%), and is biased towards genomic regions with annotated functionality (e.g. near genes and regulatory elements). Therefore, findings of this study should be interpreted with these limitations in mind. To comprehensively understand repetitive element and PMD DNAm, higher resolution approaches such as whole genome bisulfite sequencing will be necessary.

Not all CpG sites undergo dynamic changes in DNAm status throughout development. Genomic imprinting, defined as parent-of-origin specific gene expression, is typically associated with regulatory regions (promoters/enhancers) that exhibit parent-of-origin dependent DNAm. Imprinting is an evolutionary phenomenon that exists only in eutherian mammals [17], which suggests a potential important relationship between placental function and imprinting. Consistent with this, there is an enrichment for imprinted genes that are specific to the placenta [18–21]. Although our study lacks parental information, previously identified placental-specific imprinted DMRs tend to show the expected intermediate DNAm levels in TB, and to a lesser degree, in EC and SC. For example, the placental-imprinted gene, *DNMT1*, is only unmethylated at its promoter in HB, while other placental cell types are hemi-methylated. DNAm-mediated down-regulation of *DNMT1* expression has been shown in whole placental tissue [48], and our data suggest that that DNAm-mediated regulation of DNMT1 varies by placental cell population. All placental-specific imprints examined in this study showed the expected intermediate methylation for TB and CV samples, and hypomethylation for HB. The methylation patterns of HBs are consistent with an origin from fetal monocytes. The variability in the patterns of DNAm at these imprinted CpGs for mesenchymal components, EC and SC, could be from variability in the timing of erasure of these imprints. Future investigations in resolving parental-origin-specific DNAm and expression are needed. In contrast, our data suggest that common (non-placental specific) imprinted CpGs are maintained in all placental cell populations.

To address the challenges of cell composition variability in placental DNAm studies, we have generated DNAm profiles for 4 major human placental cell populations as well as enzymatically isolated STB, and assessed their utility as references for cellular deconvolution. Like other tissues [26, 37, 49], placental cell composition can be estimated with any of the commonly used deconvolution approaches. However, it was not possible to independently validate the DNAm-based cell composition estimates presented in this study with other quantitative measures of cell composition (e.g. with histology) and it is not possible to get measures on the identical sample assayed for DNAm. Instead, we validated bioinformatically estimated cell composition in cultured trophoblasts and in previously published samples that are enriched for certain populations (e.g. by enzymatically stripping away the outer layers of chorionic villi). Estimated cell composition in first trimester and term samples was also consistent with our understanding of how placental cell composition changes across gestation. The ratio of CTB:STB is relatively equal at 13 weeks gestation [32]. But as trophoblastic surface area increases as pregnancy progresses [50], at term, 90% of nuclei exist in STB and the remainder are in CTB [32]. This corresponds to a large observed increase in the eSTB component (+ 58% from first trimester), becoming the predominant cell population at term. However, we note that within the STB, nuclei are also heterogeneous in their chromatin state, where there are 4 times more transcriptionally inactive nuclei compared to active ones [51]. This property, combined with how similar CTB and STB methylation profiles are, may limit the ability to accurately estimate eSTB proportion. There were also no nRBCs estimated as present in either first trimester or term placentas, suggesting that their contribution to placental cell composition may be very small, at least in uncomplicated pregnancies. Together, these observations suggest that this approach is able to capture large relative changes, but may be imprecise when assessing smaller changes. Future studies with independent measures of cell composition, such as from histology, will be essential for assessing the accuracy of this approach, as has been done for cell deconvolution in other tissues such as adult/cord blood and brain [26, 37, 49].

There was also significant interindividual variation in cell composition that could not be fully explained by within-trimester gestational age variation, suggesting that other factors contribute to cell composition variability. In this study, we found that chronological (i.e. reported) and biological (i.e. estimated from DNAm) gestational age, sex, and ancestry were not significantly associated with cell composition. But the sample size supporting these findings was small and future studies with more appropriate power are needed to answer how much these factors play in contributing to placental cell composition variability. We also caution that the accuracy of cell composition estimates on first trimester samples relies on the degree of gestational-age dependent variation in term eSTB and nRBC reference CpGs, which could not be assessed in this study.

Another challenge to this study, and others which use a single or few marker genes/proteins to isolate /define cell populations, is addressing heterogeneity within relatively homogenous cell populations. As mentioned previously, TB contains several subtypes, such as CTB, STB and EVTs. In this study, our TB is likely mostly CTB but contains some proportion of immature precursors to the other TB subtypes, given that pan-trophoblast markers EGFR+ were used for cell isolation. HB (CD14+/CD68+) and SC (VIM+) can also able to be divided into meaningful subtypes [7]. It will be essential to placental epigenetics research to develop DNAm references for other placental cell subtypes. This will be especially important in studies on placental pathologies (and likely also in many other phenotypes), where certain TB subtypes are more affected than others, such as preeclampsia [52] and placenta accreta [53]. Associated changes in cell composition with preeclampsia may explain the finding that many CpGs with altered DNAm in preeclampsia [30] are also highly cell-specific. Cellular heterogeneity will always be a challenge when using techniques that take measurements in samples that consist of a mixed population of cells. However, cell deconvolution applied in placental DNAm studies will significantly improve interpretation of the resulting measurements and findings.

## Conclusions

This study provides a comprehensive characterization of the placental methylome at a cell-specific resolution. A major finding of this study was that many canonical placental-specific DNAm features are maintained in trophoblasts, often not observed in Hofbauer cells, and variably maintained in mesenchymal components, endothelial and stromal cells. Because samples were obtained from healthy subjects, this data serves as a reference for prioritizing the study of epigenetically regulated genomic regions. Additionally, this cell-specific reference data can be directly used to estimate placental cell composition from placental chorionic villi DNAm, which will be useful when interpreting findings from future placental DNAm studies.

## Methods

### Patient recruitment

Placental tissues were obtained with approval from the University of British Columbia / Children’s and Women’s Health Centre of British Columbia Research Ethics Board (H04–70488, H16–02280, H13–00640). Women for a scheduled C-section with a healthy term (> 37 weeks) singleton pregnancy were recruited with written informed consent at BC Women’s Hospital, Vancouver Canada. In addition, first trimester samples from elective terminations were obtained in a deidentified manner. A total of 9 first trimester (6.4–13 weeks) and 19 term (36.4–40.4 weeks) placental samples were obtained; all were screened for large chromosome abnormalities using CNV calling on the EPIC array, and found to be normal. No gross pathologies were noted.

### Tissue processing and cell isolation

Fresh term placenta samples from 3 to 4 sites were taken from the fetal side of the placental disc to avoid maternal contamination and pooled for processing. Chorionic villi samples were washed several times in 1X PBS to eliminate all traces of visible blood and physically homogenized using razor blades. For term placental samples the tissue was then incubated twice in a denuding/digestion HEPES buffer containing HBSS, Dispase, trypsin and DNase I for 30 min at 37C°, to allow the separation of most of the syncytiotrophoblast layer of the chorionic villi. The remaining tissue was then washed in HBSS media with 2% FBS (HF media) and subsequently digested using Collagenase/Hyaluronidase Digestion DMEM Buffer with DNase I, at 37oC for 1 h with vortexing every 30 min. The supernatant was collected. This is followed by a wash of the remaining cell pellet with HF media, gentle centrifugation at 4 °C for 10 min and further digestion of the cell pellet, with gentle mixing with a pipette, using of 0.25% trypsin solution for 2 min at room temp. The pellet is then washed again with HF media and digested once again with a Dispase/DNase I solution by gentle mixing with a pipette. This is followed by a final wash in HF media and filtering of the sample using first 100um and then 40um sieves to eliminate any remaining chunks of tissue. The cells are then counted and frozen in freezing media at − 80 °C until used for FACS. The freezing process eliminates a great deal of the remaining non-nucleated red blood cells.

For first trimester samples, the entire placental sample was processed after identification and removal of most of the decidual tissue. The sample was mechanically homogenized using razor blades and then digested with Collagenase/Hyaluronidase DMEM Buffer at 37 °C for 1 h. The tissue was then washed with HF media and further digested with a 0.25% trypsin solution by gentle mixing with a pipette for 2 min. The pellet is then washed again with HF media and digested once again with a Dispase/DNase I solution by gentle mixing with a pipette for 2 min. The sample was finally washed with HF media and filtered through a 40um sieve. The cell pellet was resuspended in HF and cells were counted and subsequently frozen in freezing media at -80oC until used for FACS.

To isolate human placental cell types with fluorescence-activated cell-sorting (FACS), cells were first thawed and then washed using HF media. Suspended cells in HF media were then filtered through a 40-um sieve (VWR, CA21008–949) and then counted using a hemocytometer. Trypan Blue (0.4%, Amresco, K940-100ML) was used to identify live / dead cells. A final cell solution was made at a concentration of 10 million cells per ml, which was then stained with the following antibodies purchased from eBioscience: 7-AAD (1:25, 00–6993-50), CD235a FITC (1:50, 11–9886-42), CD45 APC-eFluor780 (1:100, 47–0459-42), CD14 PE (1:50, 12–0149-42), CD34 APC (1:25, 17–0349-42), and EGFR PeCy7 (Biolegend, 1:50, 352,909). Approximately 200,000 cells for term placental samples and 125,000 cells for first trimester were obtained for each cell type using the BCCHR FACS Core equipment. DNA was extracted from cell-sorted samples and matched whole villi using Qiagen DNeasy Blood & Tissue kit (Qiagen, 69,504 / 69,506).

Enzymatically isolated Syncytiotrophoblasts (eSTB) were obtained from term villi samples using an enzymatic digestion protocol. Briefly, approximately 0.5 cc of chorionic villi were washed thoroughly several times with 1X PBS to eliminate all visible traces of blood without disrupting the tissue and then incubated for 10 min in 1 ml of Collagenase IA 1 mg/ml (Sigma). The tube was then vortexed for 30 s, if cloudy, 3 ml of Hanks Balanced salt solution (HBSS) was added to the digest letting it settle for 2 min. The supernatant containing mostly syncytiotrophoblast (STB) and some cytotrophoblast (CTB) was collected in a separate tube, the pellet was centrifugated and washed in 1X PBS before DNA extraction. This HBSS step is repeated once and all supernatant is pooled in the same tube. If the initial collagenase IA digest is not cloudy after the initial 10 min digestion, the whole villi were digested for an additional 2 min before adding the HBSS.

### Measuring DNA methylation in placental samples

DNA quality was checked using a NanoDrop ND-1000 (Thermo Scientific) as well as by electrophoresis on a 1% agarose gel. Bisulfite conversion was carried out using the EZ DNA Methylation Kit (Zymo, D5001 and D5002), before amplification and hybridization to the Infinium Methylation EPIC BeadChip (Illumina, WG-317) following the manufacturer’s protocol. An Illumina iScan reader was used to scan the chips and produce raw data files (IDATs).

### DNA methylation data processing

To assess various quality metrics, IDAT files were loaded directly into R (v3.6.1) using the *minfi* package (v1.32.0) and *ewastools* [28] (v1.6). Poor quality and unreliable probes (detection *p*-value > 0.01, bead count < 3, cross-hybridizing [54], probes with SNPs within 5 bp of the CpG site in the probe direction [54]), and probes located on sex chromosomes were removed (*n* = 109,410). Analysis was restricted to a final set of 737,050 autosome probes. All samples had high (7500–15,000 average median intensity readings in the methylated and unmethylated channels, and passed manufacturer-determined default thresholds for 17 control probes. The possibility of sample mislabelling was verified comparing reported sex and inferred sex based on X chromosome copy number (*ewastools*) [28]. Identical genotypes between matched cell-sorted and whole chorionic villi samples were verified using the EPIC array’s 59 SNP probes (*ewastools*) [28]. This genotype-check also identified a number of first trimester cell-sorted samples with evidence of maternal contamination, which were removed from further analyses (*n* = 12). Upon inspection of global DNAm patterns with PCA, we identified and removed 2 outlier samples that we suspect were contaminated with cells from other genotype-matched samples. Further details on detecting maternal contamination are described in Additional File 2. After quality control and probe filtering, noob [55] and BMIQ [56] normalization was applied to the DNAm data.

### Differentially methylated CpGs (DMCs) analysis

All analyses were conducted in R version > 3.6.1. To identify differentially methylated CpGs (DMCs), the R package *limma* [57] (v3.42.0) was used to apply CpG-wise linear models with empirical Bayes posterior variance estimators [58]. Unless otherwise stated, the “one-versus-all” approach was applied, where for each CpG, the mean DNAm of one cell type was compared to the mean of all other samples (excluding villi). DMCs were defined as those tests that were statistically significant at a bonferroni-adjusted p-value of < 0.01, and also a showed a difference in mean DNAm > 25%. For functional enrichment analysis of identified DMCs, the R package *missmethyl* (v1.20.0) was used to account for the variable number of CpGs that can be associated with each gene [59]. For testing enrichment of DMCs for various genomic features (e.g. CpG islands, promoters, enhancers) and preeclampsia-associated CpGs, chi-squared tests were applied using the base-R function *chi.sq.test*. Annotations for UCSC transcripts (e.g. promoters, introns, exons, etc.), enhancers, and CpG islands were taken from the R package *annotatr*, which downloads annotation data from UCSC directly. Significant enrichment/depletion was defined as those with a Bonferroni-adjusted *p* < 0.01. DMRs were identified using the R package *dmrcate* (v2.0.7)*,* using an FDR cutoff of < 0.01, with default settings.

### Partially methylated domains

To assess cell-specific placental DNAm in partially methylated domains (PMDs), coordinates for previously identified placental PMDs were taken from Schroeder et al. 2011 [15]. Original hg18 coordinates were mapped to hg19 using the UCSC LiftOver tool implemented in the R package *liftover* (v1.10.0). Due to differences in genomic content between the two genome versions, remapping broke up many PMD regions into smaller ones. Fifteen of these smaller “pieces” mapped to different chromosomes, so were removed from further analysis. To account for bias in array-specific coverage towards CpGs lying in promoters, CpG islands, and CpG island shores, these CpGs were removed, as previously described [15].

### Imprinted regions

Location data for imprinted regions was created by combining results from five previous human imprinting studies [18–21, 39]. A variety of approaches and technologies were used in these studies, such as whole genome bisulfite sequencing, methyl-sequencing, and Infinium 450 k methylation arrays. We took outer coordinates for overlapping regions. A final list of imprinted regions can be found in Additional File 7, Table S7.

Repetitive element mappings were determined by downloading the “rmsk” track from UCSC genome browser (hg19) and then overlapping these regions with the 850 k array CpGs. Mean DNAm was calculated by averaging over each set (Alu, LINE1, all CpGs) of CpGs for each sample. Predicted genome-wide DNAm for Alu and LINE1 CpGs was done using the Bioconductor R package *REMP* [41] (v1.8.2), using default settings.

### Public cord blood DNAm data

A curated database of cord blood cell types DNAm data ran on the 450 k methylation array was used. This data was downloaded from the R package *FlowSorted.CordBloodCombined.450 k* and noob normalized [37]. For associated analyses, the common probes from this dataset and our EPIC data were used. Heatmaps/clustering was applied using the R package *pheatmap*. Where possible, colour-blind friendly palettes were used with the R package *viridis*.

### Cellular deconvolution

Reference probes for cellular deconvolution were determined using the *pickCompProbes* function from the R package *minfi* (v1.30.0) separately for first trimester and third trimester samples. The Houseman et al. 2012 constrained projection (CP) approach was applied using implementations in the *minfi* and *EpiDISH* (v2.0.2) R packages. Other algorithms tested were robust partial correlations (RPC) and CIBERSORT (CBS), both implemented in the *EpiDISH* package. Default parameters were used for all functions, except “constraint” was set to “equality” for using the CP approach from *EpiDISH*. In silico mixtures were generated by the following procedure: 250 proportion samples were drawn from a uniform distribution between 0 and 1. These are the first 250 proportions for one cell type. Two hundred fifty additional proportions were drawn from a uniform distribution between 0 and the first proportion for the next cell type. This was repeated for a total of 6 times for 6 cell types. These 5 sets of 250 sampled proportions make up 250 in silico mixtures. Because this procedure only ensures that the first set of percentages are uniformly distributed from [0,1] and the remainder are biased towards increasingly smaller values, we repeated this entire procedure for each cell type, each time starting with a different cell type, for a total of 1500 in silico mixtures. Performance metrics to compare algorithms were computed using the r package *yardstick* (v0.0.4). Linear modelling with Bonferroni- multiple testing adjustment was done to test differences in cell composition by sex and by ethnicity. Inferred ethnicity was computed via the R package *planet* (v0.2.0) [60], and corroborated with the first 2 principal components of high density (~ 2.3 million SNPs) genotyping data

## Supplementary Information

**Additional file 1 Figure S1** Fluorescence-activated cell-sorting and immune fluorescence staining. A) Fluorescence-activated cell-sorting (FACS) workflow schematic. B-E) Immunofluorescence staining (IF) of term cell-sorted sample with known characteristic cell type markers that were not selected for in the FACS procedure. Nuclei are shown via DAPI staining (blue). Scale bars: 100 μm. B) Trophoblasts (KRT7: green, VIM: red). C) Hofbauer cells (CD68: green). D) Endothelial cells (CD31: green). E) Stromal cells (VIM: red). **Figure S2** Identifying maternal contamination. A) Total intensity over all probes from X and Y chromosomes normalized to total autosomal intensity can be used to determine sex. B) Within-donor sample-sample correlation on SNP probes. C) SNP distributions (*n* = 59 probes). D) Theoretical relationship between the average probability SNP is an outlier from the expected distribution, and maternal contamination. E) Empirically observed relationship between the average probability a SNP is an outlier, and normalized Y intensity, in male samples. Normalized Y intensity is a quantifiable measure of maternal contamination in male samples. F) Training a linear predictor of maternal contamination in male samples, then applying it to female samples. **Figure S3** Principal component (PC) associations with phenotype variables. Principal components were tested for their association with various biological and technical sample variables. Each PC was tested individually in a simple linear model with each sample variable. **Figure S4** First Trimester differentially methylated CpGs enrichment for genomic location. First trimester differentially methylated CpGs were tested for enrichment at various genomic features (e.g. CpG island, enhancers, gene transcripts, PMDs). **Figure S5** Mean DNAm for each cell type across CpGs in selected functionally-relevant genes. Average term placental cell-specific DNA methylation across select genes. Differentially methylated regions (defined as regions with a high density of differentially methylated CpGs), are highlighted with a grey background. **Figure S6** Mean DNAm for each cell type across CpGs in selected preeclampsia genes. Average term placental cell-specific DNA methylation across select genes. Differentially methylated regions (defined as regions with a high density of differentially methylated CpGs), are highlighted with a grey background. **Figure S7** Density graphs of CpGs in imprinted regions. A) Density plots (y-axis) of imprinted regions divided into those that are imprinted in more than one tissue (top) and placental-specific (bottom). The percentage of CpGs falling within 25%–75% is labelled in each plot. First trimester samples are shown. B) Maternal imprinted regions. Density of DNAm at CpGs in maternally imprinted regions. The total percentage of CpGs that have 25% - 75% DNAm are shown in each plot. C) Paternally imprinted regions. **Figure S8** DNAm at imprinted regions for specific genes. A) Cell-specific DNAm at placental-specific imprinted regions for genes FGF8 and B) FGF12 **Figure S9** DNAm at imprinted regions for specific genes. A) Cell-specific DNAm at placental-specific imprinted regions for genes JMJD1C and B) FGF14. **Figure S10** DNAm at imprinted regions for specific genes. A) Cell-specific DNAm at placental-specific imprinted regions for genes RASGRF1. **Figure S11** DNAm summarized over repetitive elements. A) Repetitive element DNA methylation. CpG sites overlapping Alu and Line 1 (L1) elements were determined using the ‘rmsk’ track from UCSC. Mean DNAm over these CpGs was calculated for each sample. B) First trimester mean DNAm across repetitive elements and all 850 k CpGs. C) REMP-predicted repetitive element DNAm in third trimester samples. **Figure S1** Comparison of cell deconvolution algorithms. A) Estimated percentage by deconvolution (y-axis) by actual percentage used to construct in silico mixtures (x-axis). Performance metrics are shown for each algorithm and cell type. RMSE, root mean squared error; R2, R squared; MAE, mean absolute error. B) Distribution of deviations from deconvolution estimates and actual percentages for in silico mixtures. The mean deviation (estimated minus actual) is labelled in each panel as text, and as the dotted vertical line. **Figure S13** Validating cell composition estimates. A) Cell deconvolution was applied to *n*=5 (labelled A-E) cultured trophoblast samples from Yuen et al. 2011 produced trophoblast-dominant samples. Trophoblast samples were treated in varying oxygen levels (1%, 8%, 20%). Half were maintained as CTB (top) and the other half was cultured for 48 h (bottom), which promotes syncytialization. B) Enzymatic treatment to separate chorionic villi samples into inner mesenchyme and outer trophoblast layer samples. Both types of samples are heterogeneous in cell composition but mesenchymal samples are enriched from endothelial and stromal cells, whereas the outer chorionic villi samples are mostly trophoblast. C) Chorionic villi was processed to isolate large stem villi, produced samples that resulted in mainly stromal in proportion compared to normally processed villi. CTB: cytotrophoblast; STB: syncytiotrophoblast. (PPTX 9610 kb)

**Additional file 2 Supplementary Methods** Detecting contamination in cell sorted samples

**Additional file 3 Table S1** Linear modelling results for differentially methylated CpGs. Results are presented for every tested CpG. First trimester results.

**Additional file 4 Table S2** Linear modelling results for differentially methylated CpGs. Results are presented for every tested CpG. Third trimester (term) results.

**Additional file 5 Table S3** DMR results for each cell type for first trimester comparisons (CSV 8251 kb)

**Additional file 6 Table S4** DMR results for each cell type for term comparisons (CSV 11093 kb)

**Additional file 7 Table S5** GO enrichment for cell DMCs. **Table S6** KEGG enrichment for cell DMCs. **Table S7** List of imprinted genes and regions from multiple studies. **Table S8** Linear modelling results of repetitive element methylation and cell type. **Table S9** 600 reference probes for cell deconvolution for term/third trimester. **Table S10** 600 reference probes for cell deconvolution for first trimester. **Table S11** Performance metrics for deconvolution algorithms on in-silico mixtures. **Table S12** Statistical testing results for cell composition versus sex, ethnicity, and gestational age. Each cell type proportion was tested against each sample variable.

## Data Availability

Cell DNAm data and supporting sample-specific information is available on GEO dataset accession number (GSE159526). Cell-specific reference CpGs for cellular deconvolution can be found in Table S5, and also online using the R package *planet* [60] (github.com/wvictor14/planet). Cell DNAm data can be explored interactively using the R shiny app *Placental Methylome Browser* [61] (https://robinsonlab.shinyapps.io/Placental_Methylome_Browser/). The hg19 and hg18 genomic coordinates were used from UCSC genome browser [62] (https://genome.ucsc.edu/).

## Abbreviations

DNAm: DNA methylation
DMC: Differentially methylated CpG
CpG: Cytosine phosphate guanine dinucleotide
PMD: Partially methylated domain
EWAS: Epigenome-wide association study
FACS: Fluorescence-activated cell-sorting
PCA: Principal components analysis
CV: Chorionic villi
TB: Trophoblasts, in this manuscript TB refers to EGFR+ cell-sorted samples from whole CV, and consists primarily of CTB
CTB: Cytotrophoblasts
eSTB: Enzymatically-separated syncytiotrophoblasts
EVT: Extravillous trophoblasts
HB: Hofbauer cells
EC: Endothelial cells
SC: Stromal cells
nRBC: Nucleated red blood cell
SNP: Single nucleotide polymorphism
CP: Constrained projection
CBS: CIBERSORT
RPC: Robust partial correlations
R^2^: R squared, proportion of variance explained
MAE: Mean absolute error
RMSE: Root mean squared error
