# Supplementary material for: Cell-specific characterization of the placental methylome
Source: BMC Genomics. 2021 Jan 6;22:6. doi: 10.1186/s12864-020-07186-6 (PMC7788826; doi:10.1186/s12864-020-07186-6)
Supplement: Supplementary file 1 — Additional file 1 Figure S1 Fluorescence-activated cell-sorting and immune fluorescence staining. A) Fluorescence-activated cell-sorting (FACS) workflow schematic. B-E) Immunofluorescence staining (IF) of term cell-sorted sample with known characteristic cell type markers that were not selected for in the FACS procedure. Nuclei are shown via DAPI staining (blue). Scale bars: 100 μm. B) Trophoblasts (KRT7: green, VIM: red). C) Hofbauer cells (CD68: green). D) Endothelial cells (CD31: green). E) Stromal cells (VIM: red). Figure S2 Identifying maternal contamination. A) Total intensity over all probes from X and Y chromosomes normalized to total autosomal intensity can be used to determine sex. B) Within-donor sample-sample correlation on SNP probes. C) SNP distributions (n = 59 probes). D) Theoretical relationship between the average probability SNP is an outlier from the expected distribution, and maternal contamination. E) Empirically observed relationship between the average probability a SNP is an outlier, and normalized Y intensity, in male samples. Normalized Y intensity is a quantifiable measure of maternal contamination in male samples. F) Training a linear predictor of maternal contamination in male samples, then applying it to female samples. Figure S3 Principal component (PC) associations with phenotype variables. Principal components were tested for their association with various biological and technical sample variables. Each PC was tested individually in a simple linear model with each sample variable. Figure S4 First Trimester differentially methylated CpGs enrichment for genomic location. First trimester differentially methylated CpGs were tested for enrichment at various genomic features (e.g. CpG island, enhancers, gene transcripts, PMDs). Figure S5 Mean DNAm for each cell type across CpGs in selected functionally-relevant genes. Average term placental cell-specific DNA methylation across select genes. Differentially methylated regions (defined as re [file 12864_2020_7186_MOESM1_ESM.pptx]

## Slide 1
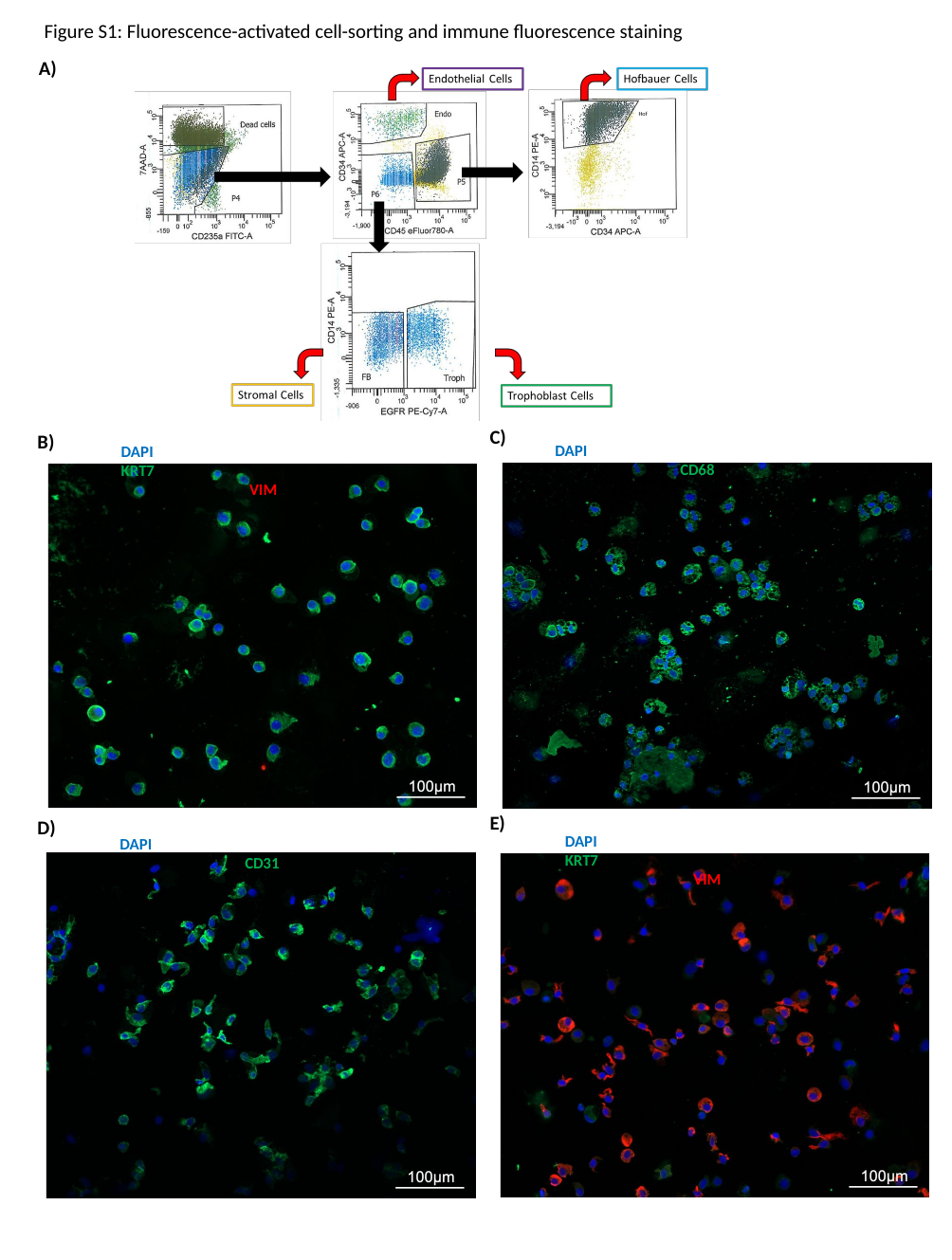

Figure S1: Fluorescence-activated cell-sorting and immune fluorescence staining
Fluorescence-activated cell-sorting and immune fluorescence staining. A) Fluorescence-activated cell-sorting (FACS) workflow schematic. B-E) Immunofluorescence staining (IF) of term cell-sorted sample with known characteristic cell type markers that were not selected for in the FACS procedure. Nuclei are shown via DAPI staining (blue). Scale bars: 100 µm. B) Trophoblasts (KRT7: green, VIM: red). C) Hofbauer cells (CD68: green). D) Endothelial cells (CD31: green). E) Stromal cells (VIM: red).
A)
Hof
C)
B)
DAPI		CD68
DAPI		KRT7		VIM
E)
D)
DAPI		KRT7		 VIM
DAPI		CD31

## Slide 2
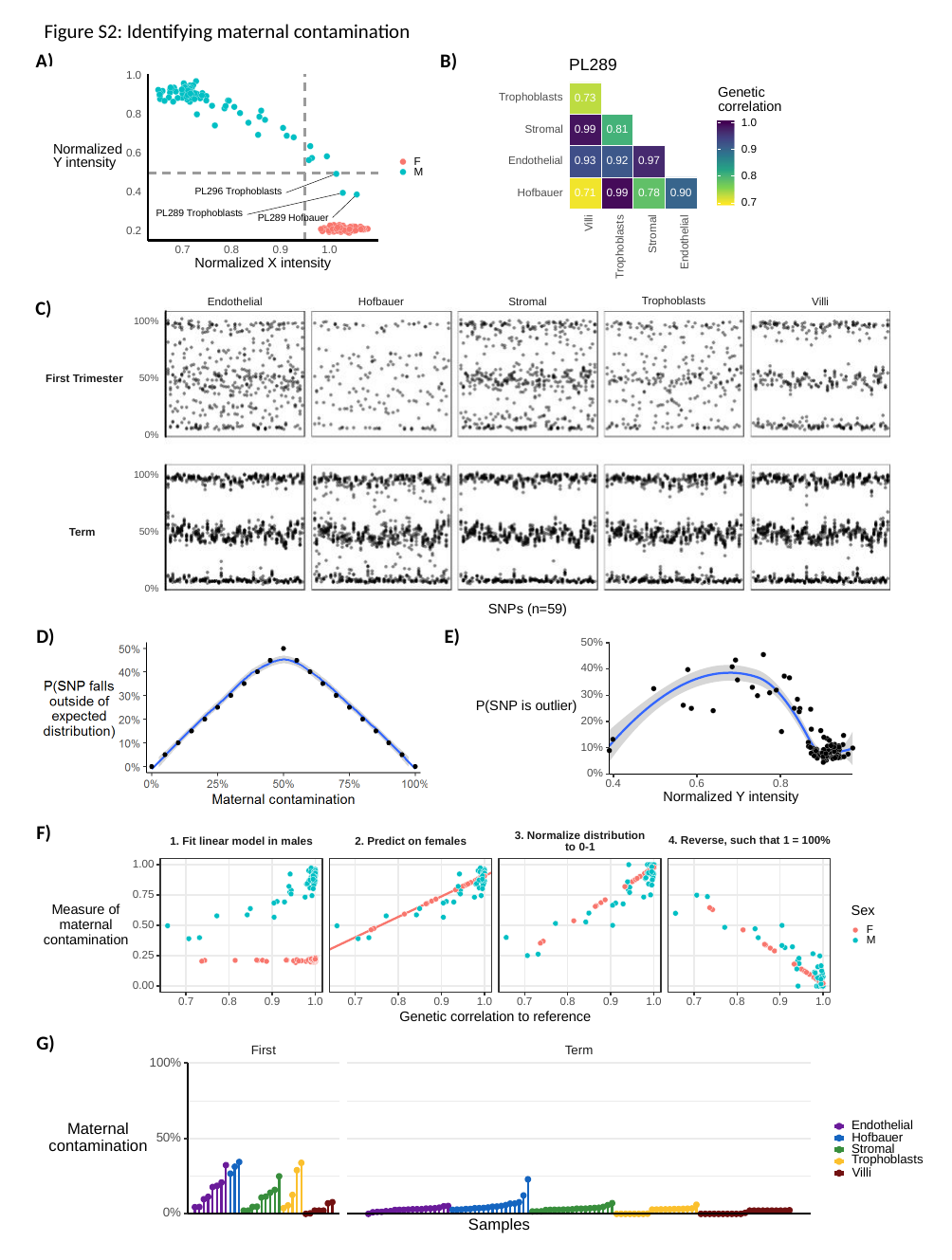

Figure S2: Identifying maternal contamination
Identifying maternal contamination. A) Total intensity over all probes from X and Y chromosomes normalized to total autosomal intensity can be used to determine sex. B) Within-donor sample-sample correlation on SNP probes. C) SNP distributions (n = 59 probes). D) Theoretical relationship between the average probability SNP is an outlier from the expected distribution, and maternal contamination. E) Empirically observed relationship between the average probability a SNP is an outlier, and normalized Y intensity, in male samples. Normalized Y intensity is a quantifiable measure of maternal contamination in male samples. F) Training a linear predictor of maternal contamination in male samples, then applying it to female samples. G) Estimated maternal contamination (y-axis) across first trimester and term samples.
B)
A)
PL289
Genetic
Trophoblasts
0.73
correlation
1.0
Stromal
0.99
0.81
0.9
0.93
0.92
0.97
Endothelial
0.8
Hofbauer
0.71
0.99
0.78
0.90
0.7
Villi
Stromal
Endothelial
Trophoblasts
1.0
0.8
Normalized
0.6
Y intensity
F
M
PL296 Trophoblasts
0.4
PL289 Trophoblasts
PL289 Hofbauer
0.2
0.7
0.8
0.9
1.0
Normalized X intensity
C)
Trophoblasts
Stromal
Hofbauer
Endothelial
Villi
100%
First Trimester
50%
0%
100%
Term
50%
0%
SNPs (n=59)
D)
E)
50%
40%
30%
P(SNP is outlier)
20%
10%
0%
0.4
0.6
0.8
Normalized Y intensity
F)
3. Normalize distribution
4. Reverse, such that 1 = 100%
2. Predict on females
1. Fit linear model in males
to 0-1
1.00
0.75
Measure of
Sex
maternal
0.50
F
contamination
M
0.25
0.00
0.7
0.8
0.9
1.0
0.7
0.8
0.9
1.0
0.7
0.8
0.9
1.0
0.7
0.8
0.9
1.0
Genetic correlation to reference
G)
First
Term
100%
Endothelial
Maternal
Hofbauer
50%
contamination
Stromal
Trophoblasts
Villi
0%
Samples

## Slide 3
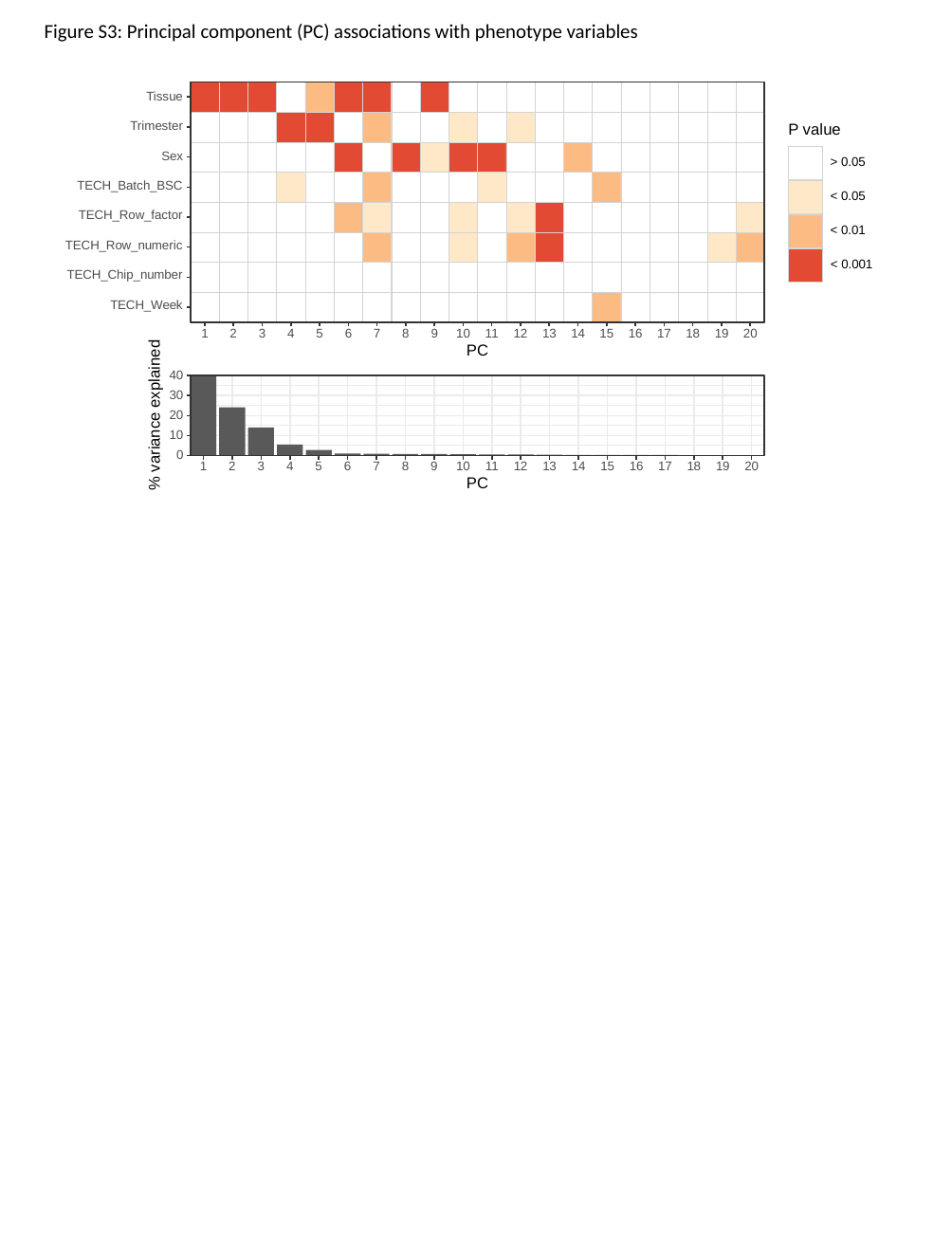

Figure S3: Principal component (PC) associations with phenotype variables
Principal component (PC) associations with phenotype variables. Principal components were tested for their association with various biological and technical sample variables. Each PC was tested individually in a simple linear model with each sample variable.
Tissue
Trimester
P value
Sex
> 0.05
TECH_Batch_BSC
< 0.05
TECH_Row_factor
< 0.01
TECH_Row_numeric
< 0.001
TECH_Chip_number
TECH_Week
3
13
6
8
9
10
15
16
18
19
20
1
2
11
12
14
17
5
4
7
PC
40
30
% variance explained
20
10
0
3
13
6
8
9
10
15
16
18
19
20
1
2
11
12
14
17
5
4
7
PC

## Slide 4
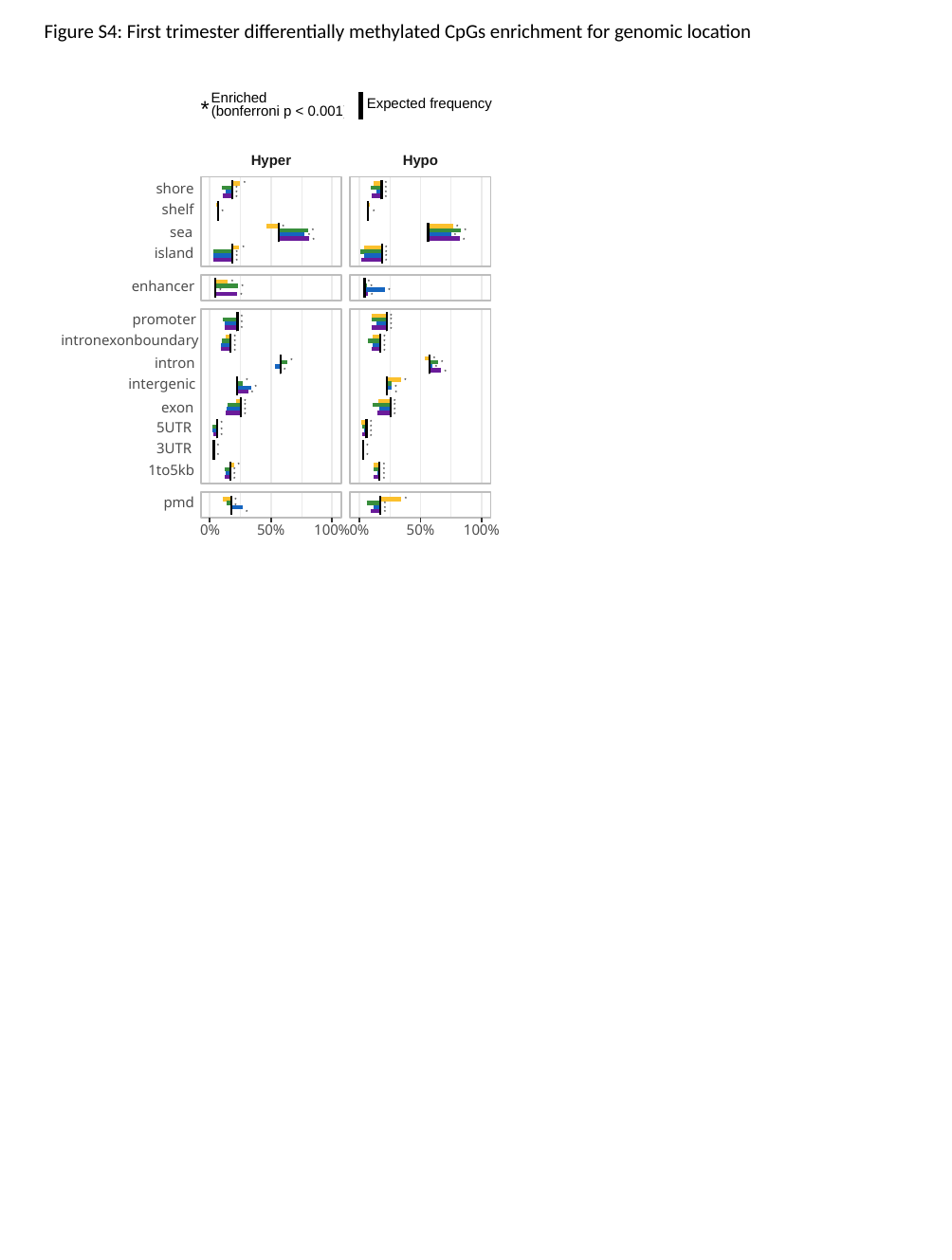

Figure S4: First trimester differentially methylated CpGs enrichment for genomic location
First trimester differentially methylated CpGs enrichment for genomic location. First trimester differentially methylated CpGs were tested for enrichment at various genomic features (e.g. CpG island, enhancers, gene transcripts, PMDs).
Enriched
Expected frequency
(bonferroni p < 0.001)
*
Hyper
Hypo
*
*
shore
*
*
*
*
*
*
shelf
*
*
*
*
sea
*
*
*
*
*
*
*
*
island
*
*
*
*
*
*
*
*
enhancer
*
*
*
*
*
*
promoter
*
*
*
*
*
*
*
intronexonboundary
*
*
*
*
*
*
*
*
*
intron
*
*
*
*
*
intergenic
*
*
*
*
*
*
*
*
exon
*
*
*
*
*
*
*
*
5UTR
*
*
*
*
*
3UTR
*
*
*
*
*
*
1to5kb
*
*
*
*
*
*
pmd
*
*
*
*
*
*
*
0%
50%
100%
0%
50%
100%

## Slide 5
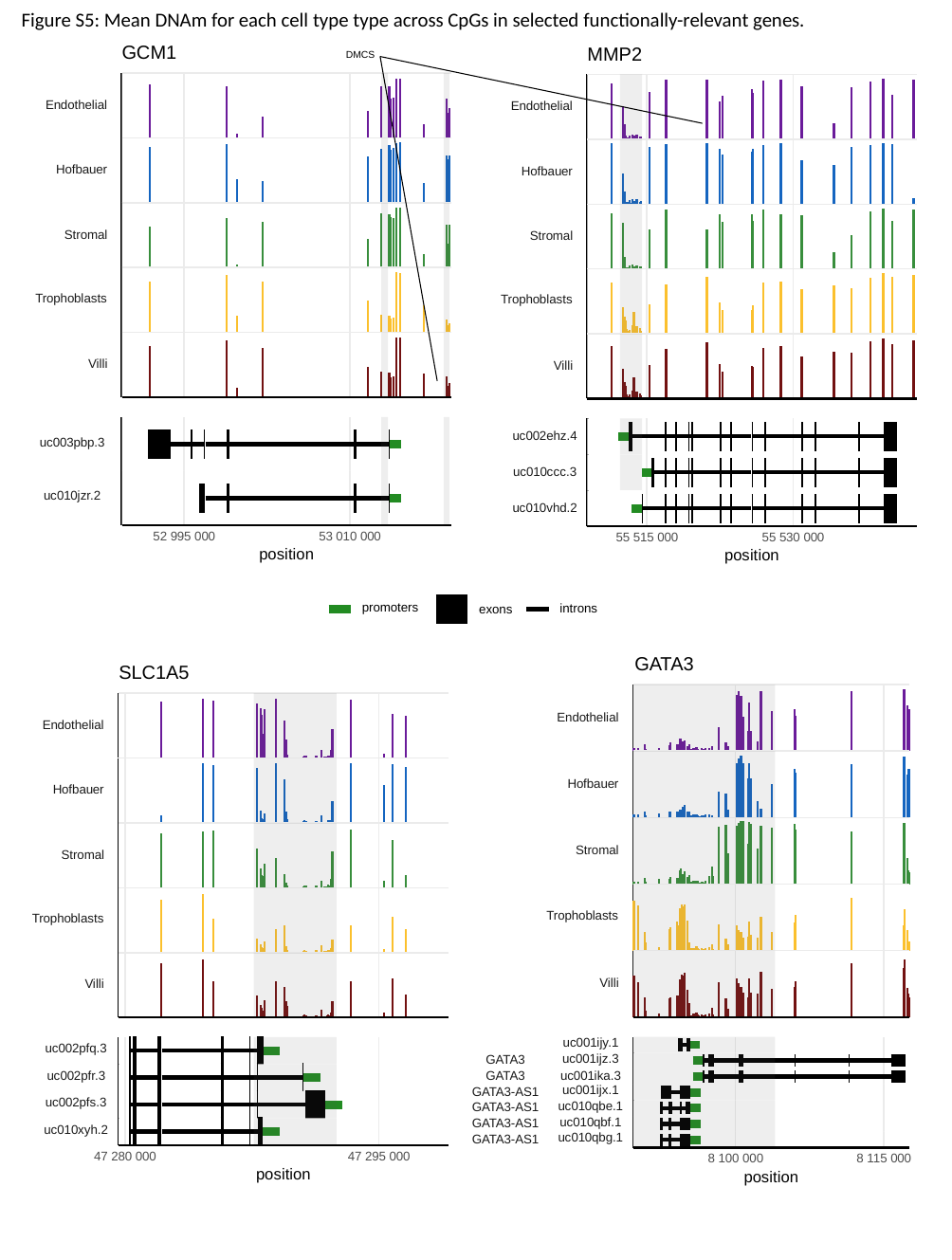

Figure S5: Mean DNAm for each cell type type across CpGs in selected functionally-relevant genes.
Mean DNAm for each cell type across CpGs in selected functionally-relevant genes. Average term placental cell-specific DNA methylation across select genes. Differentially methylated regions (defined as regions with a high density of differentially methylated CpGs), are highlighted with a grey background.
DMCS
GCM1
Endothelial
Hofbauer
Stromal
Trophoblasts
Villi
uc003pbp.3
uc010jzr.2
53 010 000
52 995 000
position
MMP2
Endothelial
Hofbauer
Stromal
Trophoblasts
Villi
uc002ehz.4
uc010ccc.3
uc010vhd.2
55 530 000
55 515 000
position
promoters
introns
exons
GATA3
Endothelial
Hofbauer
Stromal
Trophoblasts
Villi
uc001ijy.1
uc001ijz.3
GATA3
GATA3
uc001ika.3
uc001ijx.1
GATA3-AS1
uc010qbe.1
GATA3-AS1
uc010qbf.1
GATA3-AS1
uc010qbg.1
GATA3-AS1
8 100 000
8 115 000
position
SLC1A5
Endothelial
Hofbauer
Stromal
Trophoblasts
Villi
uc002pfq.3
uc002pfr.3
uc002pfs.3
uc010xyh.2
47 280 000
47 295 000
position

## Slide 6
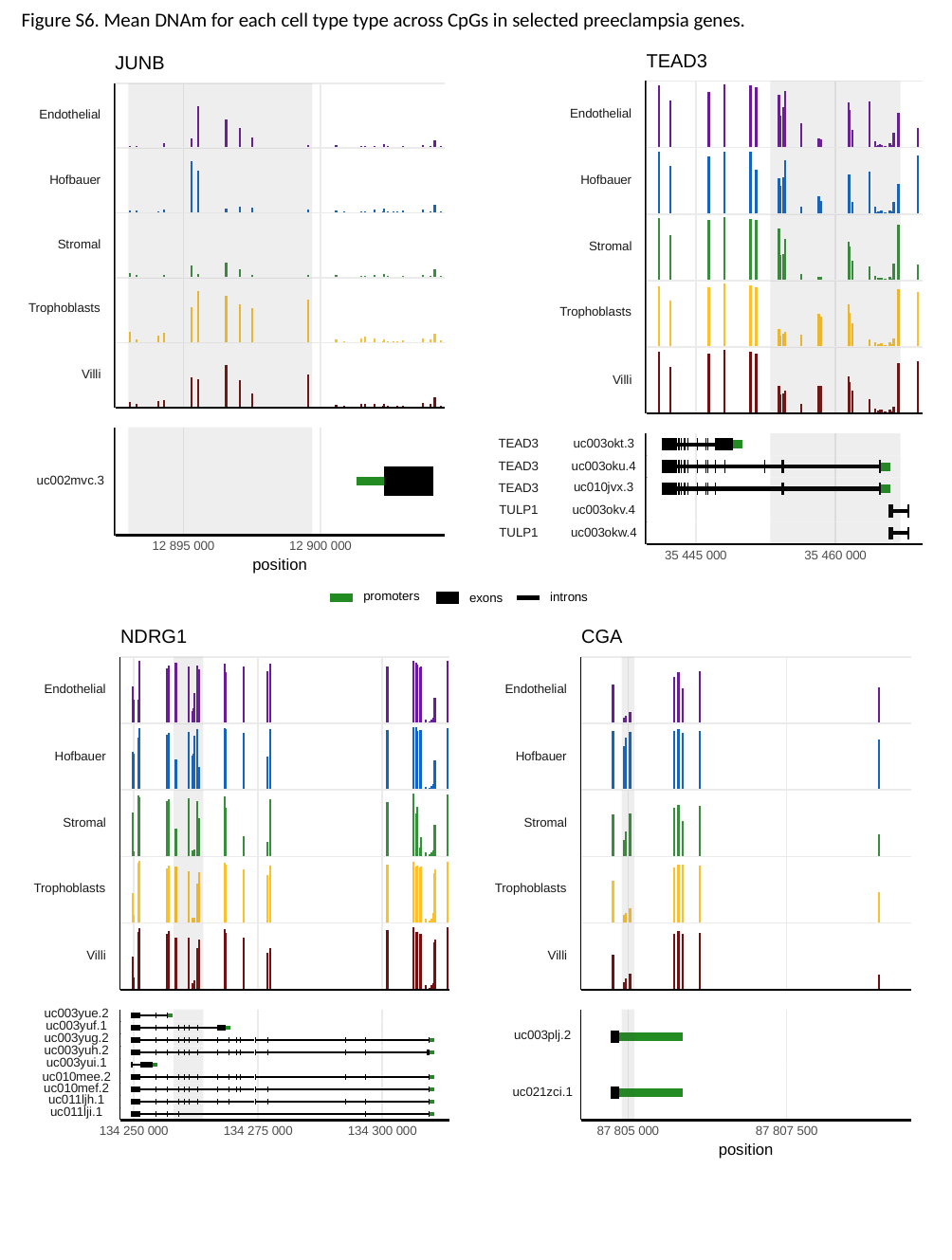

Figure S6. Mean DNAm for each cell type type across CpGs in selected preeclampsia genes.
Mean DNAm for each cell type across CpGs in selected preeclampsia genes. Average term placental cell-specific DNA methylation across select genes. Differentially methylated regions (defined as regions with a high density of differentially methylated CpGs), are highlighted with a grey background.
TEAD3
Endothelial
Hofbauer
Stromal
Trophoblasts
Villi
TEAD3
uc003okt.3
TEAD3
uc003oku.4
uc010jvx.3
TEAD3
uc003okv.4
TULP1
uc003okw.4
TULP1
35 445 000
35 460 000
JUNB
Endothelial
Hofbauer
Stromal
Trophoblasts
Villi
uc002mvc.3
12 895 000
12 900 000
position
promoters
introns
exons
NDRG1
Endothelial
Hofbauer
Stromal
Trophoblasts
Villi
uc003yue.2
uc003yuf.1
uc003yug.2
uc003yuh.2
uc003yui.1
uc010mee.2
uc010mef.2
uc011ljh.1
uc011lji.1
134 250 000
134 275 000
134 300 000
CGA
Endothelial
Hofbauer
Stromal
Trophoblasts
Villi
uc003plj.2
uc021zci.1
87 805 000
87 807 500
position

## Slide 7
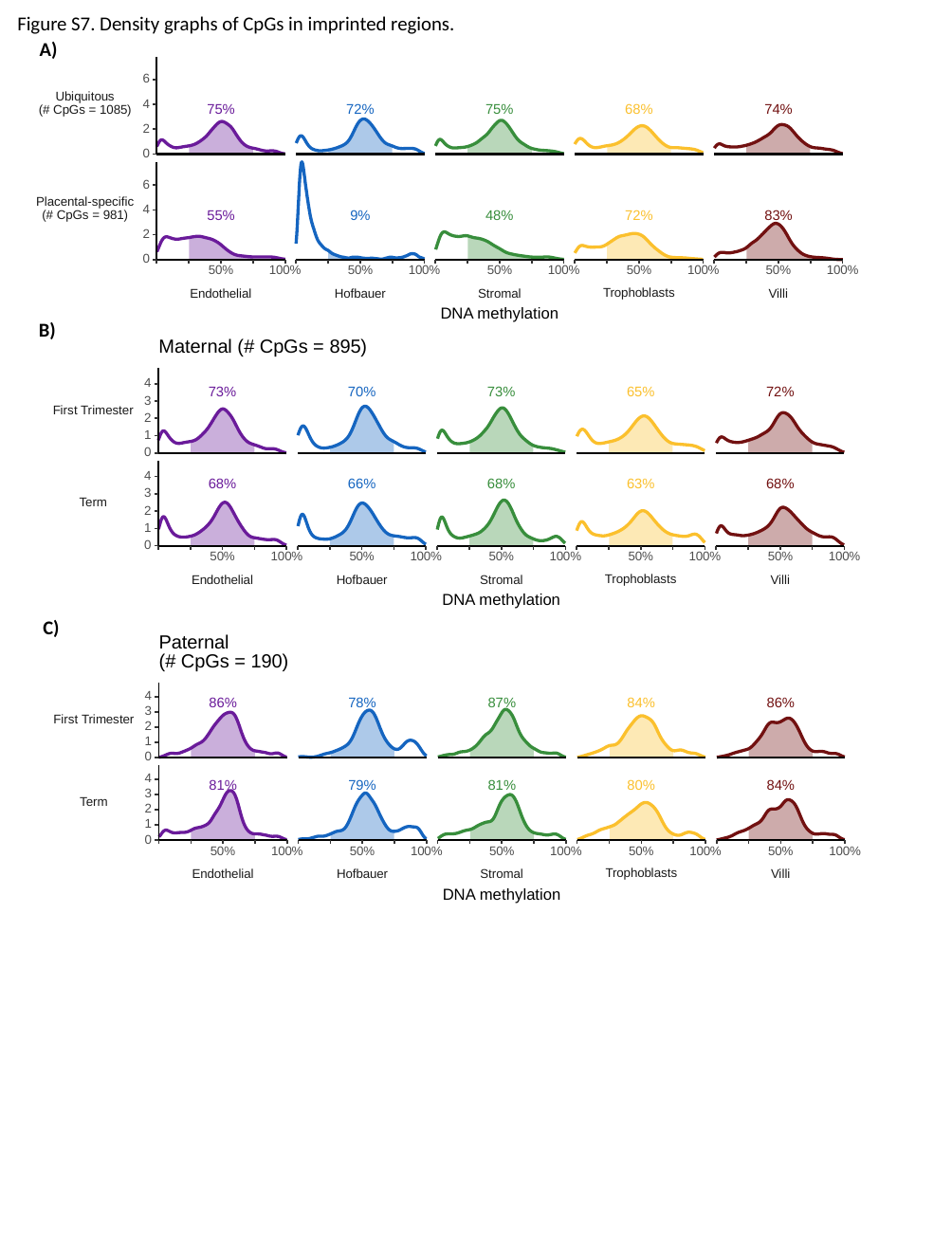

Figure S7. Density graphs of CpGs in imprinted regions.
A)
6
Ubiquitous
4
(# CpGs = 1085)
75%
72%
75%
68%
74%
2
0
6
Placental-specific
4
(# CpGs = 981)
55%
9%
48%
72%
83%
2
0
50%
100%
50%
100%
50%
100%
50%
100%
50%
100%
Trophoblasts
Stromal
Hofbauer
Endothelial
Villi
DNA methylation
Density graphs of CpGs in imprinted regions. A) Density plots (y-axis) of imprinted regions divided into those that are imprinted in more than one tissue (top) and placental-specific (bottom). The percentage of CpGs falling within 25%-75% is labelled in each plot. First trimester samples are shown. B) Maternal imprinted regions. Density of DNAm at CpGs in maternally imprinted regions. The total percentage of CpGs that have 25% - 75% DNAm are shown in each plot. C) Paternally imprinted regions.
B)
Maternal (# CpGs = 895)
4
73%
70%
73%
65%
72%
3
First Trimester
2
1
0
4
68%
66%
68%
63%
68%
3
Term
2
1
0
50%
100%
50%
100%
50%
100%
50%
100%
50%
100%
Trophoblasts
Stromal
Hofbauer
Endothelial
Villi
DNA methylation
C)
Paternal
(# CpGs = 190)
4
86%
78%
87%
84%
86%
3
First Trimester
2
1
0
4
81%
79%
81%
80%
84%
3
Term
2
1
0
50%
100%
50%
100%
50%
100%
50%
100%
50%
100%
Trophoblasts
Stromal
Hofbauer
Endothelial
Villi
DNA methylation

## Slide 8
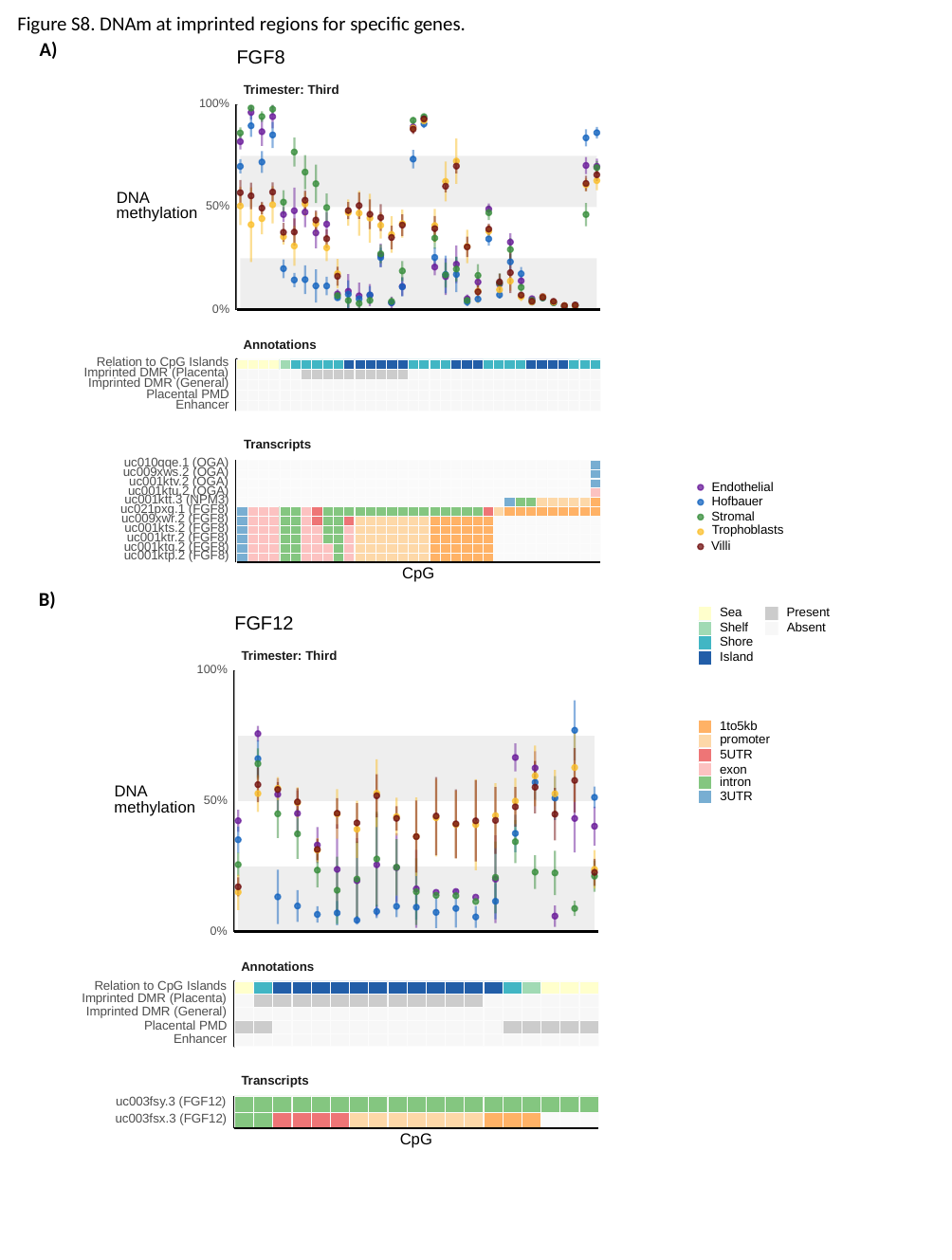

Figure S8. DNAm at imprinted regions for specific genes.
A)
FGF8
Trimester: Third
100%
DNA
50%
methylation
0%
Annotations
Relation to CpG Islands
Imprinted DMR (Placenta)
Imprinted DMR (General)
Placental PMD
Enhancer
Transcripts
uc010qqe.1 (OGA)
uc009xws.2 (OGA)
uc001ktv.2 (OGA)
uc001ktu.2 (OGA)
uc001ktt.3 (NPM3)
uc021pxg.1 (FGF8)
uc009xwr.2 (FGF8)
uc001kts.2 (FGF8)
uc001ktr.2 (FGF8)
uc001ktq.2 (FGF8)
uc001ktp.2 (FGF8)
CpG
DNAm at imprinted regions for specific genes. A) Cell-specific DNAm at placental-specific imprinted regions for genes FGF8 and B) FGF12.
Endothelial
Hofbauer
Stromal
Trophoblasts
Villi
Sea
Present
Shelf
Absent
Shore
Island
1to5kb
promoter
5UTR
exon
intron
3UTR
B)
FGF12
Trimester: Third
100%
DNA
50%
methylation
0%
Annotations
Relation to CpG Islands
Imprinted DMR (Placenta)
Imprinted DMR (General)
Placental PMD
Enhancer
Transcripts
uc003fsy.3 (FGF12)
uc003fsx.3 (FGF12)
CpG

## Slide 9
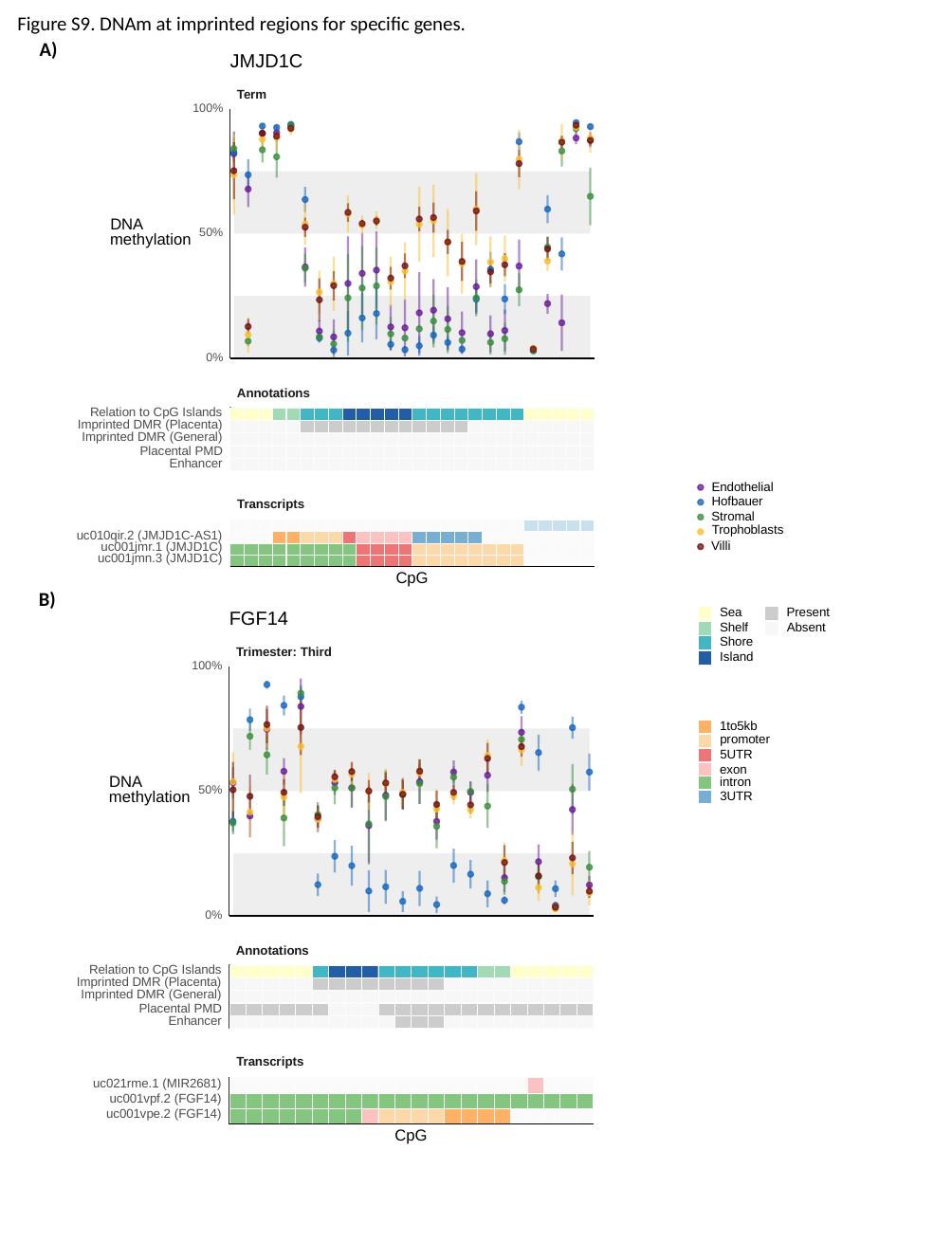

Figure S9. DNAm at imprinted regions for specific genes.
A)
JMJD1C
Term
100%
DNA
50%
methylation
0%
Annotations
Relation to CpG Islands
Imprinted DMR (Placenta)
Imprinted DMR (General)
Placental PMD
Enhancer
Transcripts
uc010qir.2 (JMJD1C-AS1)
uc001jmr.1 (JMJD1C)
uc001jmn.3 (JMJD1C)
CpG
DNAm at imprinted regions for specific genes. A) Cell-specific DNAm at placental-specific imprinted regions for genes JMJD1C and B) FGF14.
Endothelial
Hofbauer
Stromal
Trophoblasts
Villi
Sea
Present
Shelf
Absent
Shore
Island
1to5kb
promoter
5UTR
exon
intron
3UTR
B)
FGF14
Trimester: Third
100%
DNA
50%
methylation
0%
Annotations
Relation to CpG Islands
Imprinted DMR (General)
Placental PMD
Enhancer
Transcripts
uc021rme.1 (MIR2681)
uc001vpf.2 (FGF14)
uc001vpe.2 (FGF14)
CpG
Imprinted DMR (Placenta)

## Slide 10
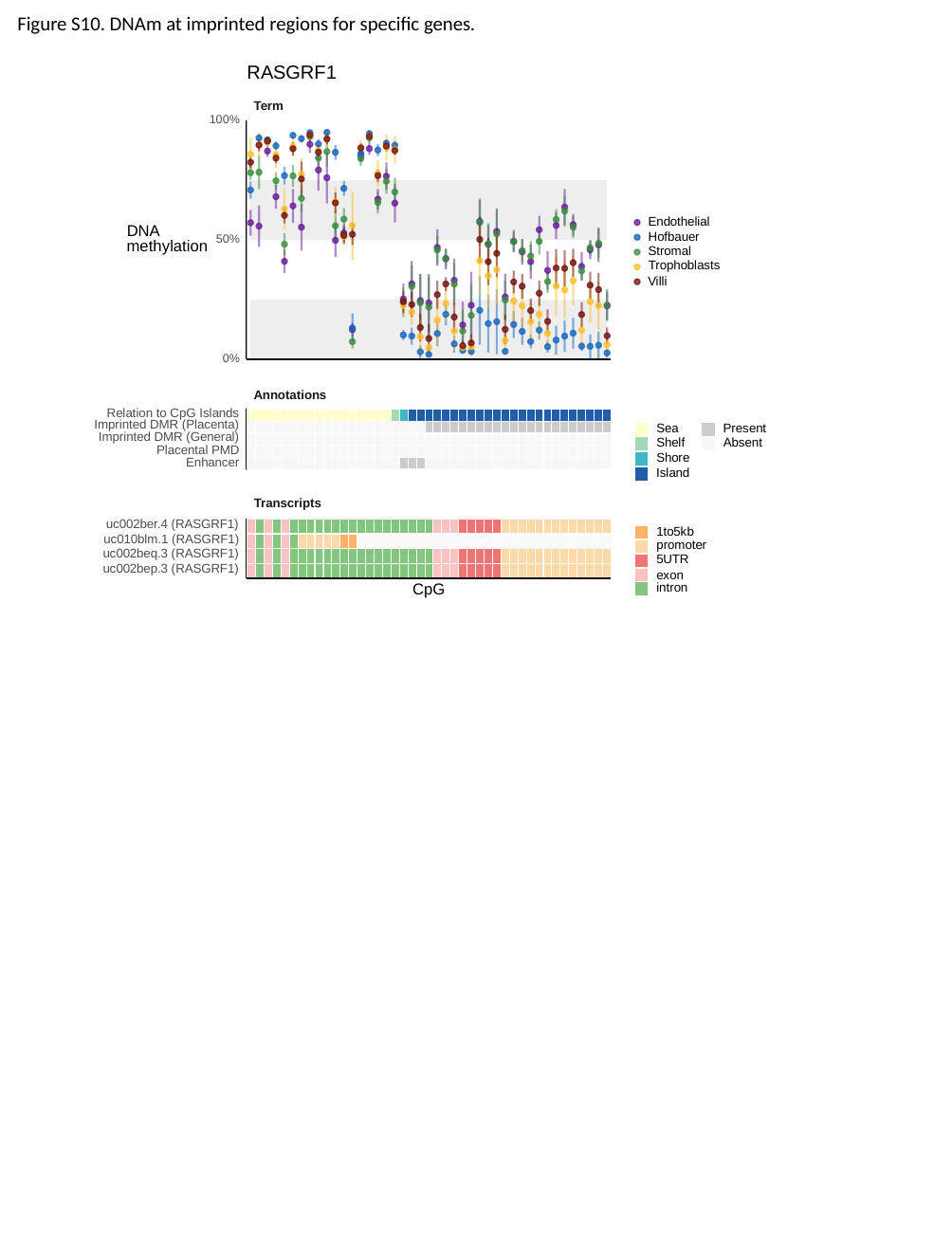

Figure S10. DNAm at imprinted regions for specific genes.
DNAm at imprinted regions for specific genes. Cell-specific DNAm at placental-specific imprinted regions for genes RASGRF1.
RASGRF1
Term
100%
Endothelial
DNA
Hofbauer
50%
methylation
Stromal
Trophoblasts
Villi
0%
Annotations
Relation to CpG Islands
Imprinted DMR (Placenta)
Sea
Present
Imprinted DMR (General)
Shelf
Absent
Placental PMD
Shore
Enhancer
Island
Transcripts
uc002ber.4 (RASGRF1)
1to5kb
uc010blm.1 (RASGRF1)
promoter
uc002beq.3 (RASGRF1)
5UTR
uc002bep.3 (RASGRF1)
exon
CpG
intron

## Slide 11
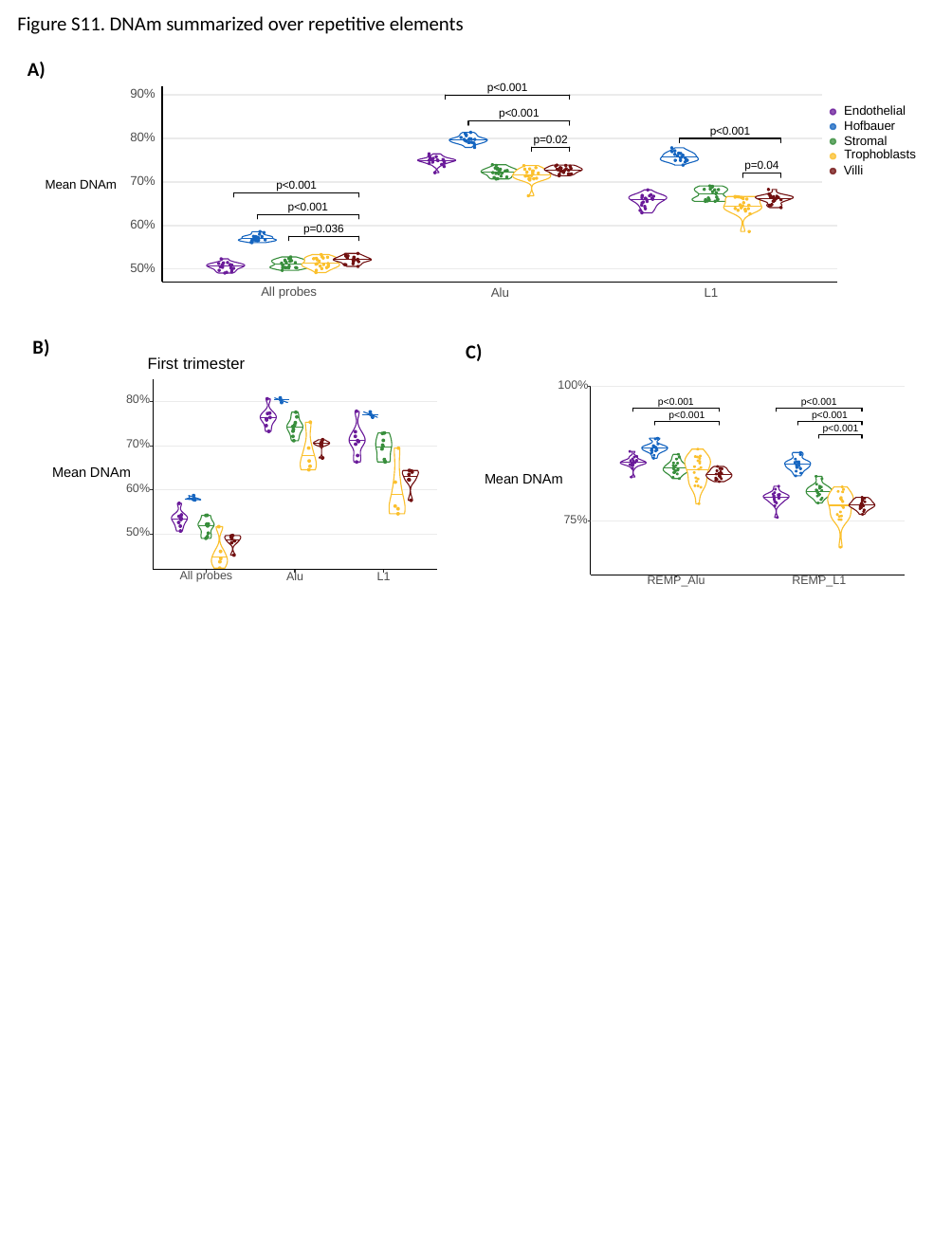

Figure S11. DNAm summarized over repetitive elements
A)
DNAm summarized over repetitive elements. A) Repetitive element DNA methylation. CpG sites overlapping Alu and Line 1 (L1) elements were determined using the ‘rmsk’ track from UCSC. Mean DNAm over these CpGs was calculated for each sample. B) First trimester mean DNAm across repetitive elements and all 850k CpGs. C) REMP-predicted repetitive element DNAm in third trimester samples.
Endothelial
Hofbauer
Stromal
Trophoblasts
Villi
p<0.001
90%
p<0.001
p<0.001
80%
p=0.02
p=0.04
70%
Mean DNAm
p<0.001
p<0.001
60%
p=0.036
50%
All probes
Alu
L1
B)
C)
First trimester
80%
70%
Mean DNAm
60%
50%
All probes
Alu
L1
100%
p<0.001
p<0.001
p<0.001
p<0.001
p<0.001
Mean DNAm
75%
REMP_L1
REMP_Alu

## Slide 12
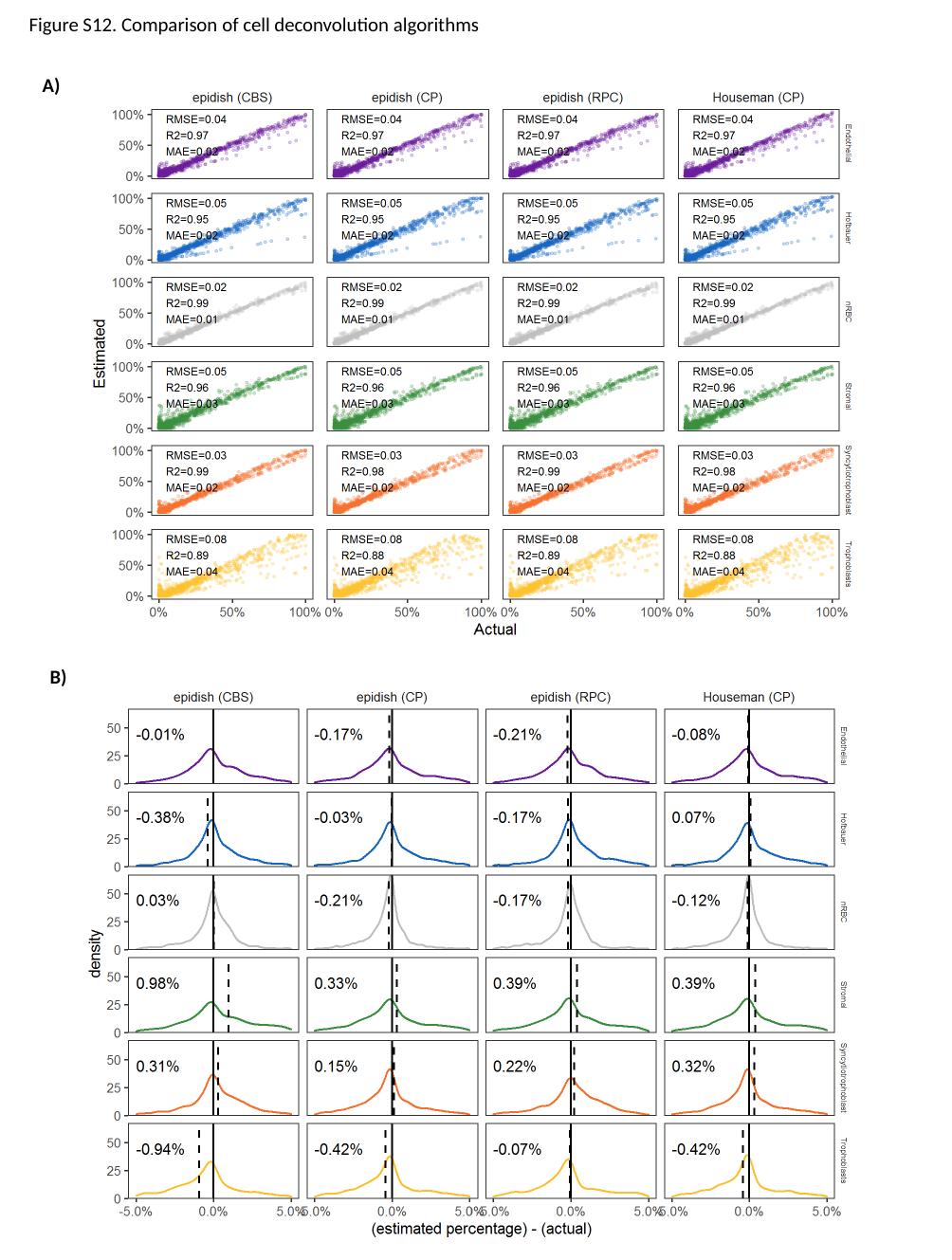

Figure S12. Comparison of cell deconvolution algorithms
Comparison of cell deconvolution algorithms. A) Estimated percentage by deconvolution (y-axis) by actual percentage used to construct in silico mixtures (x-axis). Performance metrics are shown for each algorithm and cell type. RMSE, root mean squared error; R2, R squared; MAE, mean absolute error. B) Distribution of deviations from deconvolution estimates and actual percentages for in silico mixtures. The mean deviation (estimated minus actual) is labelled in each panel as text, and as the dotted vertical line.
A)
B)

## Slide 13
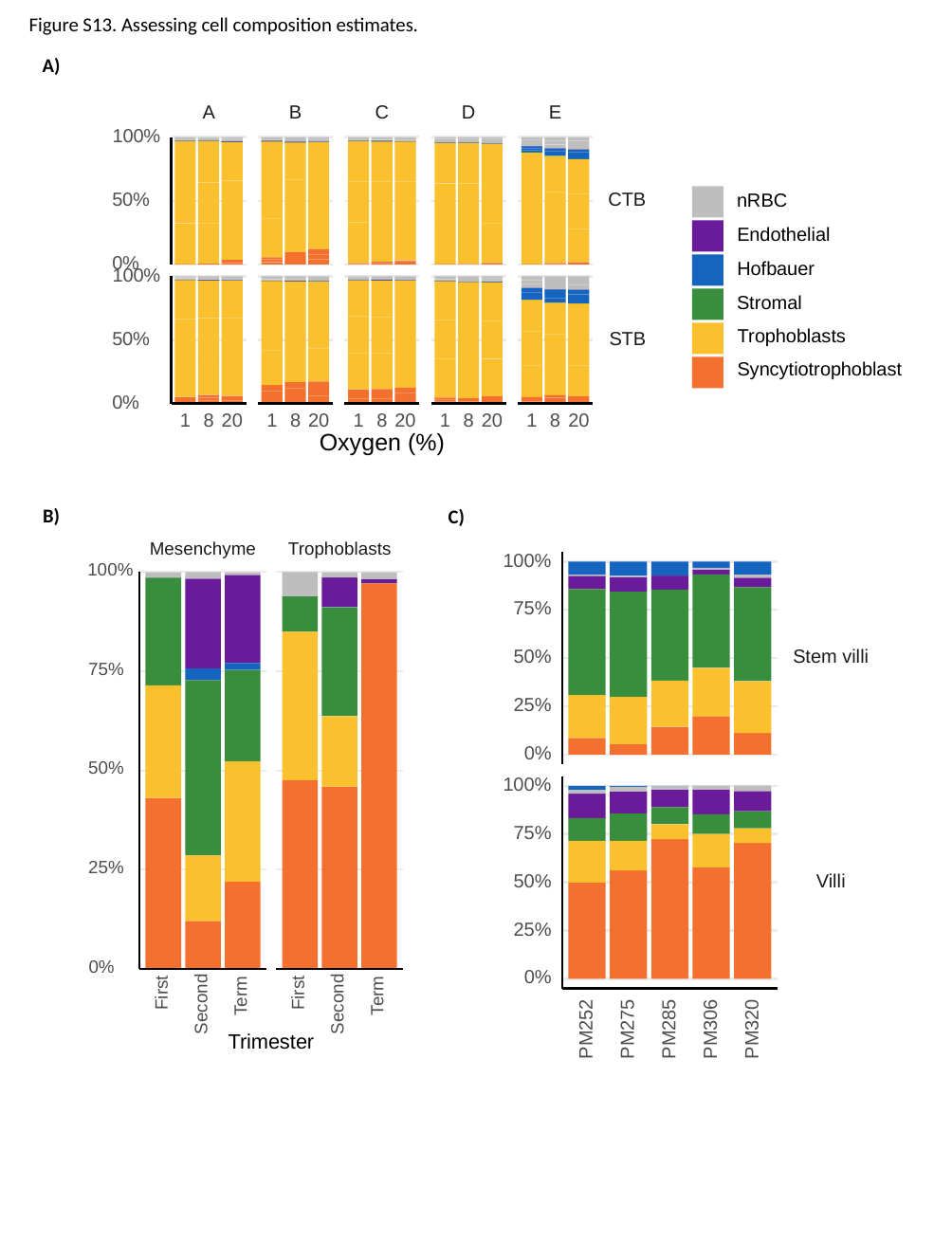

Figure S13. Assessing cell composition estimates.
Validating cell composition estimates. A) Cell deconvolution was applied to n=5 (labelled A-E) cultured trophoblast samples from Yuen et al. 2011 produced trophoblast-dominant samples. Trophoblast samples were treated in varying oxygen levels (1%, 8%, 20%). Half were maintained as CTB (top) and the other half was cultured for 48 hours (bottom), which promotes syncytialization. B) Enzymatic treatment to separate chorionic villi samples into inner mesenchyme and outer trophoblast layer samples. Both types of samples are heterogeneous in cell composition but mesenchymal samples are enriched from endothelial and stromal cells, whereas the outer chorionic villi samples are mostly trophoblast. C) Chorionic villi was processed to isolate large stem villi, produced samples that resulted in mainly stromal in proportion compared to normally processed villi. CTB: cytotrophoblast; STB: syncytiotrophoblast.
C
A
B
D
E
100%
50%
CTB
nRBC
Endothelial
0%
Hofbauer
100%
Stromal
Trophoblasts
50%
STB
Syncytiotrophoblast
0%
8
20
8
20
8
20
8
20
8
20
1
1
1
1
1
Oxygen (%)
A)
Mesenchyme
Trophoblasts
100%
75%
50%
25%
0%
First
First
Term
Term
Second
Second
Trimester
B)
C)
100%
75%
50%
Stem villi
25%
0%
100%
75%
50%
Villi
25%
0%
PM306
PM320
PM252
PM275
PM285
