# Supplementary material for: Cell-specific characterization of the placental methylome
Source: BMC Genomics. 2021 Jan 6;22:6. doi: 10.1186/s12864-020-07186-6 (PMC7788826; doi:10.1186/s12864-020-07186-6)
Supplement: Supplementary file 2 — Additional file 2 Supplementary Methods Detecting contamination in cell sorted samples [file 12864_2020_7186_MOESM2_ESM.docx]

Supplemental Methods

**Determining samples with contaminating DNA.**

There were several approaches used to determine if a sample had contamination from 1) maternal DNA, or from 2) other cell samples. First, we observed that several samples from male pregnancies had lower signal coming from Y chromosome probes then expected (Figure S2A). This suggested that these samples had contaminating DNA from a female sample, likely from contaminating maternal tissue. Many of these male samples with low Y intensity also had a lower genetic correlation to other samples from the same donor (Figure S2B). We observed that many of these suspected contaminated samples had high variation in their SNP distributions consistent with a mixing of genetically distinct samples (i.e. maternal + fetal DNA; Figure S2C).

SNP beta values measured on the EPIC array tend to fall into three distinct distributions centered around 0, 0.5, 1 corresponding to AA, AB, and BB genotypes. Deviation from these genotype peaks occurs when samples contain genetically distinct DNA molecules, for example from contaminating maternal tissue on fetally-derived placental tissue. To quantify the amount of maternal contamination in our samples, we used the R package *ewastools*, which fits a 3-component beta mixture model, where each component corresponds to one of each genotype. In addition to these 3 components, a uniform distribution is fitted which measures the probability that a SNP is an outlier to the three genotype components. This probability increases with contamination and reaches a maximum when contamination is 50%, and then decreases as the sample becomes predominantly the contaminating sample (contamination > 50%).

Consistent with this model, in male samples, the relationship between total signal from Y probes and the average probability that SNPs are outliers is an inverted “U-shape” curve. This relationship only holds true in male samples because, unlike in females, “total signal from Y probes” is related to levels of maternal contamination (Figure S2D).

Each of the described approaches measures maternal contamination. However, each approach has limitations that prevented us from applying it to the entire dataset: Low within-donor sample-sample genetic correlations can indicate that some pair of samples are genetically distinct, but do not determine which sample in these pairs is the more genetically fetal one. This approach is also a relative measure of contamination – for example, if all samples are 10% contaminated with maternal DNA, the average sample-sample genetic correlation will be around 100%. The average probability a SNP is an outlier, averaged over all 59 SNPs, increases linearly when maternal contamination is below 50%, but samples that have higher than 50% maternal contamination will start showing decreasing probabilities, making it difficult to discern between samples that are mostly maternal versus mostly fetal. Using normalized Y intensity addresses the limitations of the previous two approaches: it has a unidirectional relationship with increasing maternal contamination and represents an absolute measure of maternal contamination. However, the relationship of Y intensity with maternal contamination is only observable in male samples. To address this constraint, we constructed a maternal contamination predictor by regressing normalized total Y intensity onto the genetic correlation to of a matched reference sample, where “normalized total Y intensity” is our linear measure of maternal contamination (Figure S2F). We picked reference samples based showing evidence of low maternal contamination each of the three measures described above. For nearly all sets of samples from the same donor, the reference sample chosen was the whole placental villi. Next, we applied this linear predictor on female samples to gain equivalent measures of maternal contamination. To aid interpretability, we constrained predicted values to 0 and 1, corresponding to 0% and 100% contaminated.
